# Perspectives on socio-ecological studies in the Northern and Southern Hemispheres

**DOI:** 10.1057/s41599-023-01545-w

**Published:** 2023-02-20

**Authors:** Alejandro Huertas Herrera, Mónica D. R. Toro-Manríquez, Cristian Lorenzo, María Vanessa Lencinas, Guillermo Martínez Pastur

**Affiliations:** 1 Centro de Investigación en Ecosistemas de la Patagonia (CIEP), Coyhaique, Chile; 2 Centro Austral de Investigaciones Científicas (CADIC CONICET), Ushuaia, Argentina; 3 Universidad Nacional de Tierra del Fuego (UNTDF), Ushuaia, Argentina

**Keywords:** Social policy, Environmental studies, Geography

## Abstract

Socio-ecology studies the relationships between human activities and natural systems and their importance in management and public policy. Our objective was to analyse how published papers in countries with a high Human Development Index (HDI) perform socio-ecological studies and compare them between the Northern and Southern Hemispheres. To do this, we used the Scopus platform as a source for searching and obtaining scientific papers about socio-ecological studies conducted in countries from the Northern and Southern Hemispheres. We calculated the number (*n*) of papers published per year and classified them using the main subject areas of the SCImago Journal & Country Rank database. Then, we analysed whether papers included specific recommendations for natural system management, nature conservation, policies or governance structures, or science in general. Besides, we studied whether the papers addressed socio-ecological studies related to flora and fauna and from what specific group of organisms or systems. Data were compared using the chi-square (*χ*^2^) test (Pearson *p* < 0.005). A total of 467 papers were analysed, where 34% were from the Southern Hemisphere (mainly Argentina, Australia, Chile, and South Africa) and 66% from the Northern Hemisphere (mainly the USA, Canada, and Spain). The Northern Hemisphere (mainly North America and Europe) played a major role in the socio-ecological knowledge exchange than the Southern Hemisphere (South America and Africa). The results showed socio-ecological studies focused mainly on generating management recommendations in social and environmental science fields. The number of studies coming from the Northern Hemisphere was significantly higher than those from the Southern Hemisphere. Most of them were conducted at a local level (e.g., watersheds or human settlements) in three different systems (i) terrestrial (e.g., forests or grasslands), (ii) freshwater (e.g., rivers or streams) and (iii) marine (e.g., coastlines or seas). Most of the studies (70%) were conducted in production systems, where the majority included livestock (mainly bovine) and aquatic fisheries (e.g., salmon, artisanal coastal fishing, or trout). Most vegetation papers (65%) were on native forests. Papers on wildlife made up 30% of all animal-related studies, with mammals, birds, and marine invertebrates (such as collars) being the most extensively researched species. This work highlighted the socio-ecological approach that was used in the analysed countries with greater HDI to develop management options for natural systems.

## Introduction

Socio-ecological systems are more closely tied to geography rather than only economic indicators. Their approach aims to understand patterns of human relationships with natural systems (Morzillo et al., 2015; McKay et al., 2020) and their importance in management and public policy formation (Orach and Schlüter, 2016; Sala and Torchio, 2019). Socio-ecology analyses the management of natural systems by social actors and organizations, and the rules, social norms and conventions underlying this management (Arnaiz-Schmitz et al., 2018; www.ipbes.net). The socio-ecological approach is inherently complex (Ekbia and Evans, 2009; Paveglio et al., 2013; Martínez-Fernández et al., 2021) and incorporates various subject areas and spatial scales (Bode et al., 2016; Barnes and Nel, 2017; De Vos et al., 2019), such as natural resource management practices and public policies (Ostrom, 2009; Domptail and Easdale, 2013; Morzillo et al., 2015; Mee et al., 2016). This approach analyses the alignment of scientific knowledge with the implementation of policies, whereby countries reveal national and international commitments between human activities and natural system balance (Norgaard et al., 2009; Shilomboleni and De Plaen, 2019; De Vos et al., 2019; Williams et al., 2020).

Academic journals are proxies for the knowledge that societies gradually generate from natural systems (Palla et al., 2015), and there is a scholarly consensus about the relationship between economic development and the scientific knowledge produced by a country (King et al., 2007; Balland et al., 2020; Angrist et al., 2021). This relationship responds to the so-called Matthew effect that describes the concept of accumulated advantage, which is reflected in many social aspects (Merton, 1968). According to this effect, countries with high social and economic development that are home to the most prestigious scientists and institutions are expected to generate more academic knowledge (e.g., scientific papers in socio-ecology) than underdeveloped ones. Moreover, in the face of humankind issues such as climate change and carbon emissions, developed countries can exemplify the trend in the stewardship of human-nature balance (Russo et al., 2019; Pye et al., 2020). However, it is not clear if the geographic location (e.g., North vs. South Hemisphere) also influences the production of scientific academic knowledge of the more developed countries, which could have a differential effect on the real application of useful results produced by socio-ecology studies to improve management, human well-being, and nature conservation. Differences between the Northern and Southern Hemispheres could be related to the economic disparity between the two regions (Goddard, 1969), as was observed by Lau (2006), Jha and McCawley (2011), and De la Escosura (2015, 2021) in relation to well-being, socioeconomic development, and policy.

However, the distinction between the northern and southern countries usually refers to the social, economic, and political divisions between nations with high gross national incomes per capita and those with lower incomes (Kowalski, 2020). Therefore, the classification of the North or South is usually based on economic factors and the standard of living of its citizens rather than its geographic location (Shariff, 1997); for example, the Global North, which includes North America, Europe, and North Asia, as well as Oceania nations like Australia and New Zealand. However, the socio-ecology approach may not respond entirely to economic concepts, such as the Global North or Global South, but to integrating social and natural systems. Corals, for example, are an important natural component for societies in Oceania (e.g., Australia, New Zealand) and North America (e.g., the United States), but not in Europe (excluding overseas territories). Another example is the agricultural production related to some ecosystems that occur in both hemispheres. For instance, the Mediterranean (e.g., vineyards), which is comparable to socio-ecological systems in countries from South America (e.g., Argentina, Chile), Africa (South Africa), Oceania (e.g., Australia), North America (e.g., the United States), and Europe (e.g., France, Spain). Hence, a geographic North and South notion, as opposed to the global North and South, fits better the socio-ecological approach.

Therefore, our objective was to analyse how countries with high Human Development Index (HDI) perform socio-ecological studies and to evaluate differences between the Northern and Southern Hemispheres. Our specific questions were: (i) Is there a predominant main subject area that occurs in developed countries in both hemispheres? Or is there a difference between hemispheres? (ii) What is the exchange of socio-ecological studies between both hemispheres? (iii) What are the main recommendations (e.g., management, conservation, public policies) derived from socio-ecological studies? Are there different recommendations associated with different hemispheres or countries? (iv) What are the predominant natural systems (e.g., marine, freshwater or terrestrial) and spatial scales (e.g., local, regional, national) analysed in each hemisphere? (v) What kind of nature (e.g., flora and fauna) are mostly included in the socio-ecological studies of each hemisphere? With this in mind, we expected that this analysis of scientific papers on socio-ecology generated in high HDI countries could reveal if there were differences between the Northern and Southern Hemispheres regarding some characteristics (subject area, exchange, recommendations, natural systems, spatial scales, kind of nature), highlighting the focus until now and the main gaps that need to be filled.

## Methodology

### Searching strategy

We used the Scopus platform (www.scopus.com) as a source for searching and collecting scientific studies. We searched for the papers using four keywords. The first three words focused on the concept of socio-ecology and its potential combinations, and the fourth keyword was the name of the country we were looking for. We wrote the keywords in the singular (e.g., socio-ecological system) to obtain more results, and we omitted the “social-ecology” term in the search to avoid articles that misuse the concept, although this term appears in many selected papers (for more details, see Colding and Barthel, 2019). In this sense, the structure of the query string for our search was: ((TITLE-ABS-KEY (“Socio-ecology*“) OR TITLE-ABS-KEY (“*Socio-ecological system*“) OR TITLE-ABS KEY (“*Socioecological system*“)) and (TITLE- ABS-KEY (“Country*“)). We surveyed all papers to get the largest possible annual list of studies, finishing the search on October 28, 2021.

### Selection strategy

Once the search results were displayed, we reviewed each paper by country and selected those linked to socio-ecological studies in the Northern and Southern Hemispheres. Selection criteria for countries were based on the Human Development Index (HDI) and its components. The HDI is a metric developed by the United Nations (UNDP, 2020a) (https://www.un.org/en/) that provides dimensions of social and economic development worldwide and we used it to identify countries with more expected scientific production. Many authors prefer this index to represent people’s quality of life and the economic standard of living in countries worldwide (e.g., Furlan and Mariano, 2021; Birkmann et al., 2022), and it is useful to understand some of these countries’ social and environmental prospects. For example, patterns of social behaviours may become habitual in high HDI countries, such as conserving certain aspects of nature. HDI classifies countries according to three critical dimensions (UNDP, 2020a): (i) long and healthy life (e.g., life expectancy at birth), (ii) knowledge (e.g., mean years of schooling), and (iii) a decent standard of living (e.g., gross national income per capita-purchasing power parity). Globally, 66 countries have very high HDIs, with 36 of them being European (*n* = 66). We selected countries with the highest HDI and categorized them by hemisphere and continent (Annex 1). We found 53 countries with high HDI in the five continents (Asia = 8, Oceania = 2, Africa = 2, South America = 3, North America = 2, Europe = 36). The average number of countries per continent was 9 and in order to avoid European overrepresentation, we only included a maximum of countries per continent which doubled the average. Because of this, we selected half of these European countries (*n* = 18), considering the order of the HDI ranking from highest to lowest, with Norway being the highest HDI and France at the limit. Countries near the Equator line were not selected because we assumed they share characteristics with the Northern and Southern Hemispheres (e.g., Singapore). For the African continent, only one country (South Africa) is a developed country according to HDI; therefore, we included Botswana, the African country with the highest medium HDI, to achieve a better representation of this continent.

**Fig. 1:**
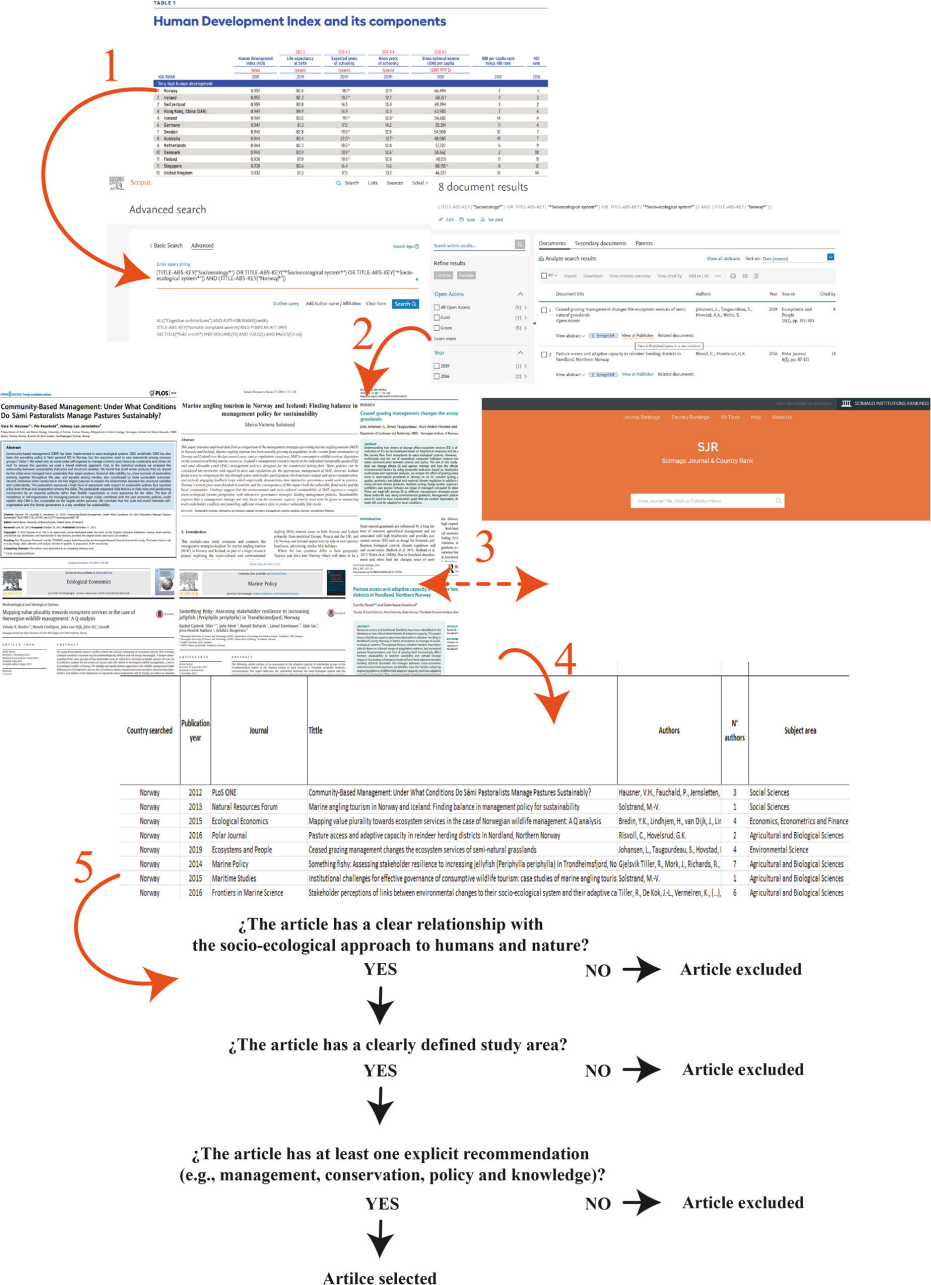
A diagram depicting how the papers chosen for review were selected. The numbers indicate each stage for the inclusion and exclusion of articles. 1 = selection of countries based on the Human Development Index (HDI); 2 = searching in Scopus (https://www.scopus.com/) for the articles of the selected countries based on the HDI (https://hdr.undp.org/); 3 = download of articles, analysis and review of their content, and identification of the research category based on Scimago (https://www.scimagojr.com/); 4 and 5 = tabulation of general information and evaluation of its relevance.

The selection strategy concerning socio-ecological studies followed these criteria (Fig. 1): (i) the studies must have a clear relationship with the socio-ecological approach to humans and nature, excluding papers related to the biological behaviour of the species (e.g., social processes of primates, dolphins, mongoose), (ii) the studies must have a clearly defined study area (e.g., country, region, water basin, city), excluding papers that present just generalities of the socio-ecological system approach, and (iii) studies must have at least one explicit recommendation (e.g., management, conservation, policy and knowledge), excluding those entirely descriptive papers.

### Data obtention

For all the papers reviewed (see Annex 2), we evaluated their multiple characteristics defining if an article deals or not with every category of each analysed characteristic. For the main subject areas, we used those defined by the “SCImago Journal & Country Rank database” (www.scimagojr.com): (i) Agricultural and Biological Sciences, (ii) Arts and Humanities, (iii) Decision Sciences, (iv) Earth and Planetary Sciences, (v) Economics, Econometrics and Finance, (vi) Environmental Sciences, (vii) Medicine, and (viii) Social Sciences. Likewise, we analysed whether the papers had specific recommendations or not for (i) Management of natural systems, (ii) Conservation of nature, (iii) Policies or governance structures, and (iv) Scientific-general knowledge (e.g., modelling approaches). We also evaluated as exclusive categories the three main spatial scales (e.g., local, regional, and national) and the three different predominant natural systems (e.g., marine, freshwater, and terrestrial systems).

### Data analysis

We calculated the quantity (*n*) of papers published each year to evaluate the timeline of socio-ecology papers. We also estimated the number of observations in YES or NO classes for each category of subject areas and recommendations, expressed in absolute frequencies and frequencies relative to the total (in percentage). To assess statistical differences among Countries and between hemispheres in the number of papers produced for some subject area or recommendation, we used chi-square (*χ*^2^) test (Pearson *p* < 0.005). The significance value followed the suggestion of Benjamin et al. (2018) for frequentist statistics. However, we consider countries with greater than or equal to 3 papers to better interpret the results. We used the R package Circlize version 0.4.13 (Gu et al., 2014) to create a chord diagram and show the proportion of the spatial scale and the different predominant natural systems. Finally, we calculated and graphed the number of papers (total *n* of all countries) that addressed socio-ecological studies related to flora and fauna.

## Results

**Fig. 2:**
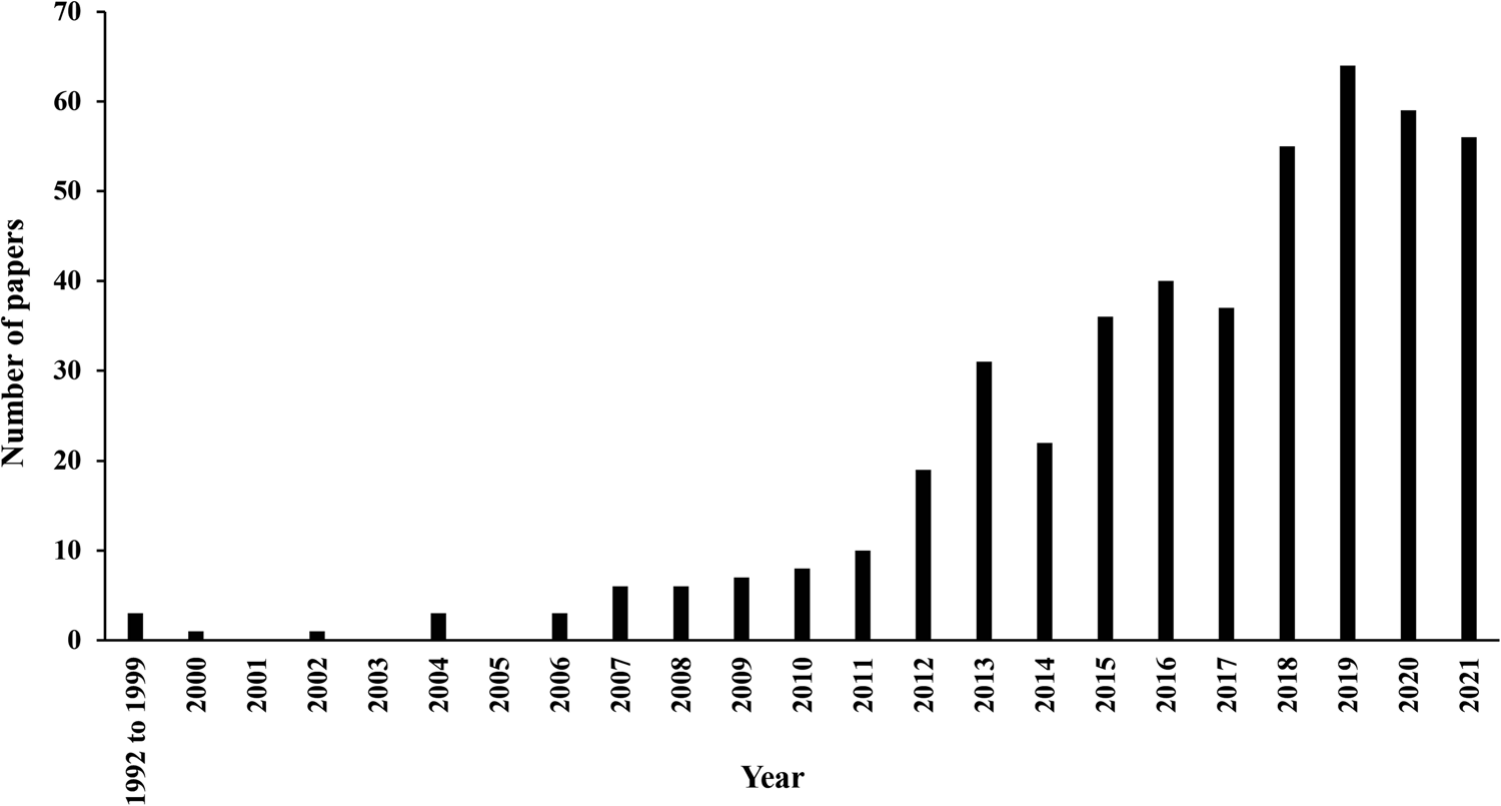
Number of studies about socio-ecology between 1992 and 2021 (*n* = 467 papers). For more details, see Annex 1.

In the analysed dataset, the earliest socio-ecological articles were published in the 1990s (Fig. 2). The first paper with a socio-ecological approach, “Environmental exploitation and social structure in prehistoric southeast Spain”, was published in the *Journal of Mediterranean Archaeology* in 1992 (see Annex 2). The second, “Socioecology of the drug problem”, appeared in the journal *Gesundheitswesen* in 1998. The third, “Coarse woody debris in riparian zones: Opportunity for interdisciplinary interaction”, was published in the *Journal of Forestry* in 1998. However, since 2006 the socio-ecological approach became more widely used, exceeding 20 papers per year after 2013 (~44 papers per year).

**Fig. 3:**
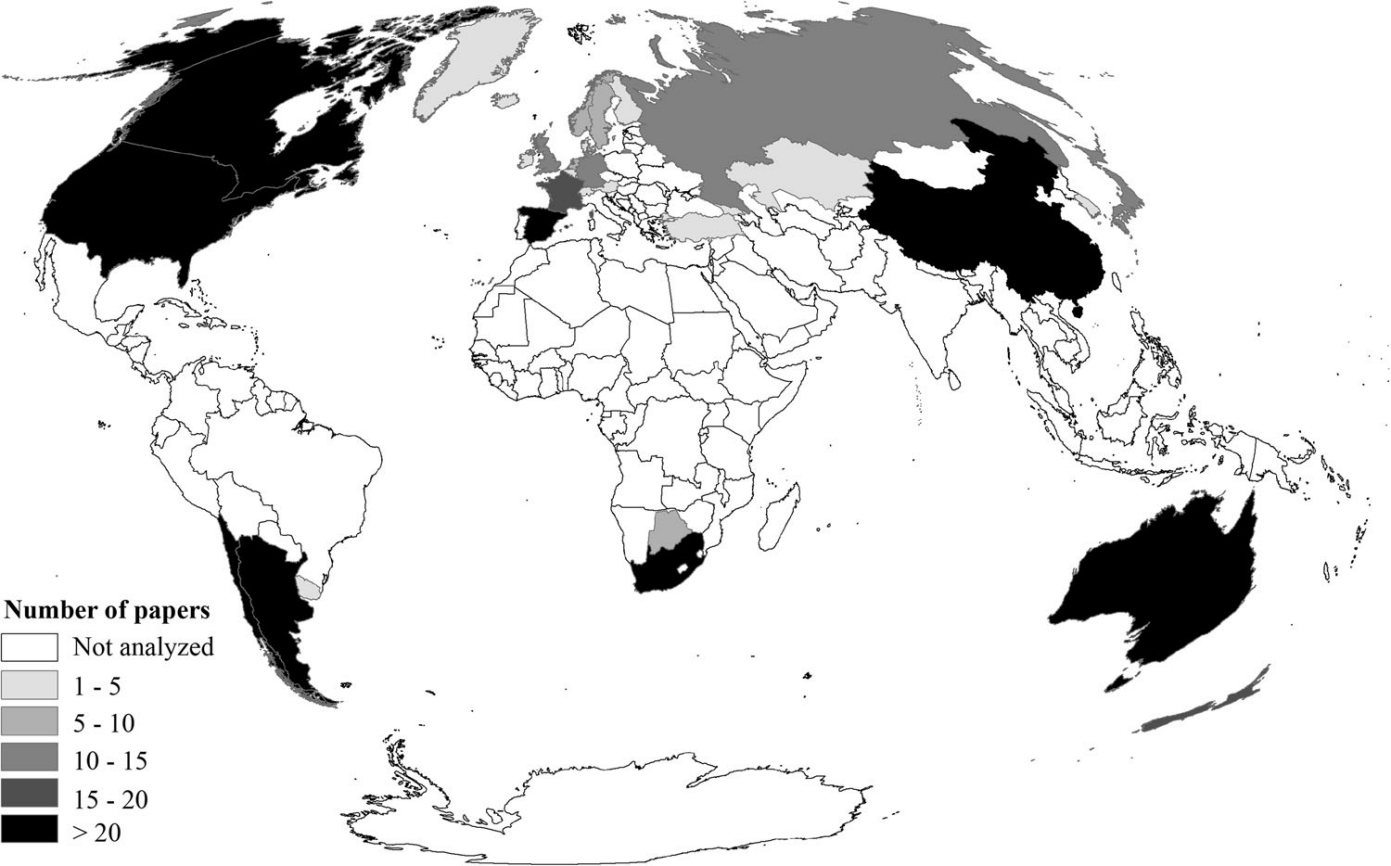
Countries with the highest number of socio-ecological scientific papers (*n* = 467 papers).

**Fig. 4:**
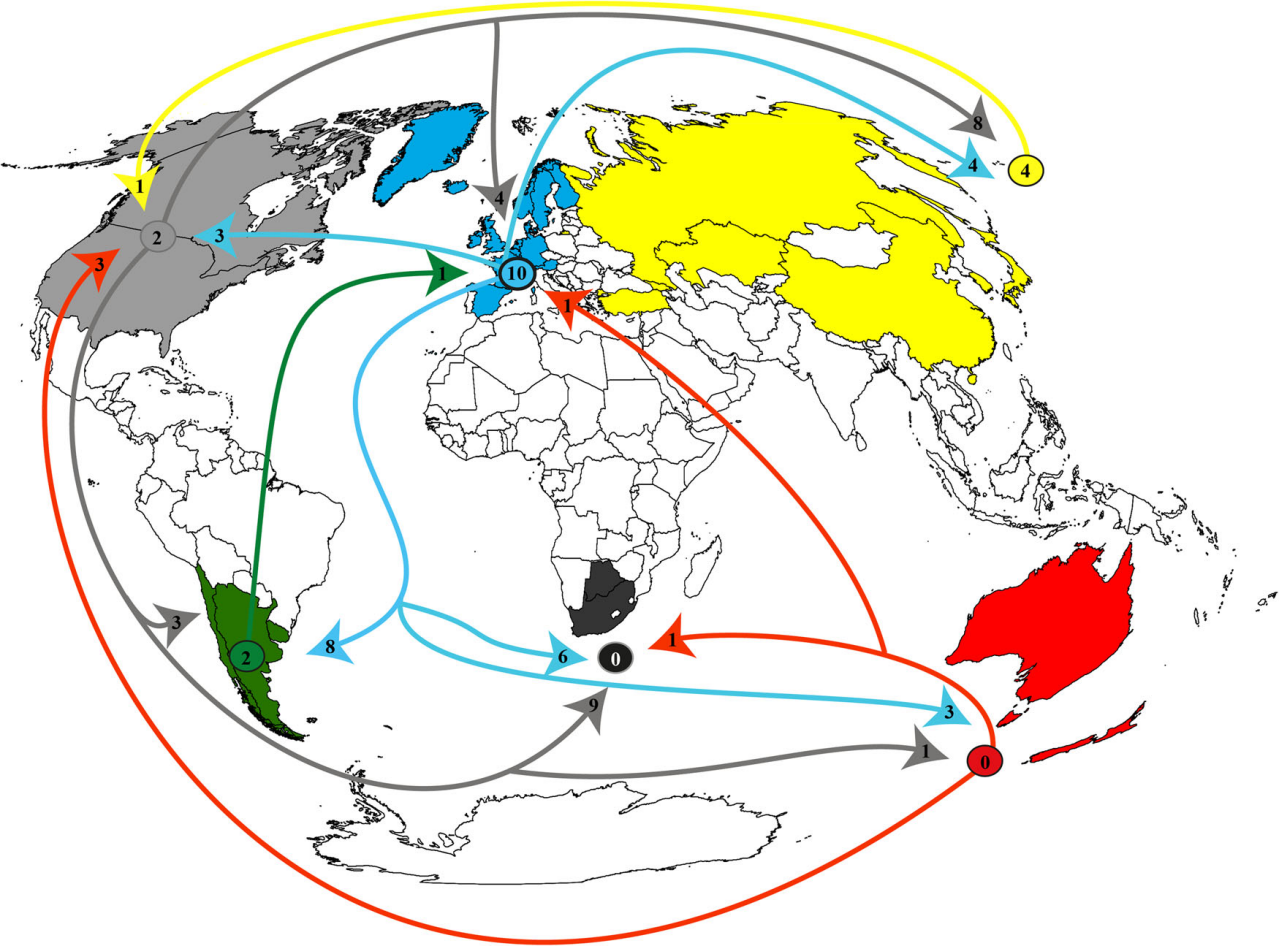
Exchange of socio-ecological knowledge. The coloured circles show the studies (*n* papers) trajecting within continents. The coloured arrows show the number of studies (*n* papers) trajecting between the continents, indicating the origin (i.e., first author´s affiliation) and destination (i.e., location of the study’s development) of the socio-ecological knowledge in both hemispheres.

We found 737 papers that somehow dealt with socio-ecology, of which we selected 63% of the studies (*n* = 467 papers) considering the three criteria of relevance that we established in the methodology section. Concerning geographical origin, we found socio-ecological studies mainly coming from North American and European countries. Geographically, institutions based in Southern Hemisphere countries produced 34% of the selected papers, with an average of 9.4 (±SD 7.1) papers per country (160 papers from 2002 to 2021), corresponding 58 to South America, 39 to Africa, and 63 to Oceania. The countries with the most papers in the Southern Hemisphere were Australia (*n* = 47), Chile (*n* = 35), South Africa (*n* = 32), and Argentina (*n* = 21). The Northern Hemisphere produced 66% of socio-ecological papers, with an average of 14.6 (±SD 15.2) papers per country (307 papers from 1992 to 2021), corresponding 93 to North America, 127 to Europe and 87 to Asia. The countries with the most papers in the Northern Hemisphere were the United States (*n* = 69), China (*n* = 53), Spain (*n* = 44), Canada (*n* = 24), and France (*n* = 20). Figure 3 shows analysed countries, classified by the number of socio-ecological studies, highlighting in black those countries with more than 20 papers in the 1992–2021 period (Argentina, Australia, Chile and South Africa in the Southern Hemisphere, and the USA, Canada, Spain and China in the Northern Hemisphere). The first author’s affiliation differences from the country where the study was conducted occurred in 74 articles (Fig. 4, Annex 3). Overall, the Northern Hemisphere exchanged more socio-ecological knowledge with the Southern Hemisphere (Fig. 4), mainly from North America and Europe to Africa and South America. Besides, Europe is the continent that has the most studies (10 papers) on the trajectory within continents, followed by North America (2 papers) and South America (2 papers). See Annex 3 for detailed country-by-country trajectories.

Considering the countries with 3 or more papers, the subject areas that presented the greatest number of papers were Social Sciences and Environmental Sciences, followed in decreasing order by Decision Sciences, Agricultural and Biological Sciences, Economics, Econometrics and Finance, Arts and Humanities, and Earth and Planetary Sciences. The Northern Hemisphere presented trends similar to the total, while in the Southern Hemisphere, Social Sciences, Decision Sciences, Arts and Environment were the ones with the highest number of papers (70 or more), Agricultural and Biological Sciences, Economics, Econometrics and Finance they had a medium amount (between 36 and 45 papers), and Medicine had the least amount (only 7) (Table 1). The analysis using the *χ*^2^ test, and the subject areas (Table 1), showed that there was a significant difference in the number of papers among countries in almost all subject areas studied, except for Decision Sciences (*χ*^2^ = 31.88; df = 22; *p* = 0.079) and Earth and Planetary Sciences (*χ*^2^ = 39.78; df = 31; *p* = 0.012). Between hemispheres, the differences were significant for Arts and Humanities (*χ*^2^ = 23.34; df = 1; *p* < 0.001), Agricultural and Biological Sciences (*χ*^2^ = 16.1; df = 1; *p* < 0.001), Environmental Sciences (*χ*^2^ = 18.51; df = 1; *p* < 0.001), Economics, Econometrics, and Finance (*χ*^2^ = 8; df = 1; *p* = 0.004), where the number of articles studied in these areas was notably higher in the Northern Hemisphere than in the Southern Hemisphere (Table 1).

**Table 1:** Number of observations expressed in absolute and relative frequencies to the total in percentage (data in parentheses) based on the socio-ecological papers by subject areas (Scopus thematic categories) per Country and Hemisphere (*χ*^2^ = chi-square test, df = degree of free, *p* value at <0.005).

	Social sciences		Arts and humanities		Agricultural and biological sciences		Environmental sciences		Economics, econometrics and finance		Decision sciences		Earth and planetary sciences		Medicine	
	No	Yes	No	Yes	No	Yes	No	Yes	No	Yes	No	Yes	No	Yes	No	Yes
*Northern Hemisphere*	86 (19)	208 (46)	228 (50.4)	66 (14.6)	153 (33.9)	141 (31.2)	120 (26.6)	174 (38.5)	189 (41.8)	105 (23.2)	153 (33.9)	141 (31.2)	235 (52)	59 (13.1)	279 (61.7)	15 (3.3)
Norway	1 (0.2)	7 (1.6)	3 (0.7)	5 (1.1)	6 (1.3)	2 (0.4)	4 (0.9)	4 (0.9)	5 (1.1)	3 (0.7)	1 (0.2)	7 (1.6)	6 (1.3)	2 (0.4)	8 (1.8)	0 (0.0)
Ireland	1 (0.2)	2 (0.4)	1 (0.2)	2 (0.4)	3 (0.7)	0 (0.0)	2 (0.4)	1 (0.2)	3 (0.7)	0 (0.0)	2 (0.4)	1 (0.2)	3 (0.7)	0 (0.0)	2 (0.4)	1 (0.2)
Switzerland	0 (0.0)	3 (0.7)	2 (0.4)	1 (0.2)	0 (0.0)	3 (0.7)	1 (0.2)	2 (0.4)	1 (0.2)	2 (0.4)	1 (0.2)	2 (0.4)	3 (0.7)	0 (0.0)	3 (0.7)	0 (0.0)
Germany	2 (0.4)	9 (2.0)	8 (1.8)	3 (0.7)	6 (1.3)	5 (1.1)	2 (0.4)	9 (2.0)	6 (1.3)	5 (1.1)	6 (1.3)	5 (1.1)	8 (1.8)	3 (0.7)	8 (1.8)	3 (0.7)
Sweden	1 (0.2)	6 (1.3)	6 (1.3)	1 (0.2)	2 (0.4)	5 (1.1)	1 (0.2)	6 (1.3)	4 (0.9)	3 (0.7)	2 (0.4)	5 (1.1)	5 (1.1)	2 (0.4)	6 (1.3)	1 (0.2)
Netherlands	5 (1.1)	1 (0.2)	5 (1.1)	1 (0.2)	4 (0.9)	2 (0.4)	1 (0.2)	5 (1.1)	5 (1.1)	1 (0.2)	3 (0.7)	3 (0.7)	5 (1.1)	1 (0.2)	6 (1.3)	0 (0.0)
Finland	1 (0.2)	2 (0.4)	3 (0.7)	0 (0.0)	1 (0.2)	2 (0.4)	1 (0.2)	2 (0.4)	2 (0.4)	1 (0.2)	1 (0.2)	2 (0.4)	1 (0.2)	2 (0.4)	3 (0.7)	0 (0.0)
United Kingdom	3 (0.7)	9 (2.0)	11 (2.4)	1 (0.2)	10 (2.2)	2 (0.4)	3 (0.7)	9 (2.0)	5 (1.1)	7 (1.6)	5 (1.1)	7 (1.6)	10 (2.2)	2 (0.4)	12 (2.7)	0 (0.0)
Belgium	0 (0.0)	3 (0.7)	1 (0.2)	2 (0.4)	3 (0.7)	0 (0.0)	1 (0.2)	2 (0.4)	1 (0.2)	2 (0.4)	1 (0.2)	2 (0.4)	3 (0.7)	0 (0.0)	3 (0.7)	0 (0.0)
Canada	2 (0.4)	22 (4.9)	12 (2.7)	12 (2.7)	11 (2.4)	13 (2.9)	10 (2.2)	14 (3.1)	21 (4.7)	3 (0.7)	14 (3.1)	10 (2.2)	20 (4.4)	4 (0.9)	21 (4.7)	3 (0.7)
United States	26 (5.8)	43 (9.5)	53 (11.7)	16 (3.5)	55 (12.2)	14 (3.1)	48 (10.6)	21 (4.7)	54 (12)	15 (3.3)	41 (9.1)	28 (6.2)	51 (11.3)	18 (4)	67 (14.8)	2 (0.4)
Austria	0 (0.0)	3 (0.7)	3 (0.7)	0 (0.0)	1 (0.2)	2 (0.4)	0 (0.0)	3 (0.7)	0 (0.0)	3 (0.7)	2 (0.4)	1 (0.2)	2 (0.4)	1 (0.2)	3 (0.7)	0 (0.0)
Japan	4 (0.9)	10 (2.2)	11 (2.4)	3 (0.7)	11 (2.4)	3 (0.7)	11 (2.4)	3 (0.7)	6 (1.3)	8 (1.8)	8 (1.8)	6 (1.3)	13 (2.9)	1 (0.2)	14 (3.1)	0 (0.0)
Spain	4 (0.9)	40 (8.9)	37 (8.2)	7 (1.6)	6 (1.3)	38 (8.4)	5 (1.1)	39 (8.6)	27 (6.0)	17 (3.8)	27 (6.0)	17 (3.8)	41 (9.1)	3 (0.7)	41 (9.1)	3 (0.7)
France	8 (1.8)	12 (2.7)	16 (3.5)	4 (0.9)	12 (2.7)	8 (1.8)	11 (2.4)	9 (2.0)	13 (2.9)	7 (1.6)	11 (2.4)	9 (2.0)	13 (2.9)	7 (1.6)	20 (4.4)	0 (0.0)
Russia	6 (1.3)	5 (1.1)	9 (2.0)	2 (0.4)	11 (2.4)	0 (0.0)	7 (1.6)	4 (0.9)	10 (2.2)	1 (0.2)	7 (1.6)	4 (0.9)	9 (2.0)	2 (0.4)	9 (2.0)	2 (0.4)
China	22 (4.9)	31 (6.9)	47 (10.4)	6 (1.3)	11 (2.4)	42 (9.3)	12 (2.7)	41 (9.1)	26 (5.8)	27 (6.0)	21 (4.7)	32 (7.1)	42 (9.3)	11 (2.4)	53 (11.7)	0 (0.0)
*Southern Hemisphere*	50 (11.1)	108 (23.9)	88 (19.5)	70 (15.5)	113 (25)	45 (10)	98 (21.7)	60 (13.3)	122 (27)	36 (8)	74 (16.4)	84 (18.6)	119 (26.3)	39 (8.6)	151 (33.4)	7 (1.6)
Australia	12 (2.7)	35 (7.7)	20 (4.4)	27 (6.0)	37 (8.2)	10 (2.2)	24 (5.3)	23 (5.1)	41 (9.1)	6 (1.3)	18 (4)	29 (6.4)	26 (5.8)	21 (4.7)	42 (9.3)	5 (1.1)
New Zealand	3 (0.7)	13 (2.9)	9 (2.0)	7 (1.6)	13 (2.9)	3 (0.7)	12 (2.7)	4 (0.9)	12 (2.7)	4 (0.9)	8 (1.8)	8 (1.8)	14 (3.1)	2 (0.4)	16 (3.5)	0 (0.0)
Chile	17 (3.8)	18 (4)	15 (3.3)	20 (4.4)	27 (6.0)	8 (1.8)	29 (6.4)	6 (1.3)	29 (6.4)	6 (1.3)	25 (5.5)	10 (2.2)	30 (6.6)	5 (1.1)	35 (7.7)	0 (0.0)
Argentina	5 (1.1)	16 (3.5)	13 (2.9)	8 (1.8)	12 (2.7)	9 (2.0)	12 (2.7)	9 (2.0)	14 (3.1)	7 (1.6)	7 (1.6)	14 (3.1)	20 (4.4)	1 (0.2)	21 (4.7)	0 (0.0)
Botswana	1 (0.2)	6 (1.3)	7 (1.6)	0 (0.0)	4 (0.9)	3 (0.7)	2 (0.4)	5 (1.1)	6 (1.3)	1 (0.2)	1 (0.2)	6 (1.3)	4 (0.9)	3 (0.7)	6 (1.3)	1 (0.2)
South Africa	12 (2.7)	20 (4.4)	24 (5.3)	8 (1.8)	20 (4.4)	12 (2.7)	19 (4.2)	13 (2.9)	20 (4.4)	12 (2.7)	15 (3.3)	17 (3.8)	25 (5.5)	7 (1.6)	31 (6.9)	1 (0.2)
Total	136(30.1)	316(69.9)	316(69.9)	136(30.1)	266(58.9)	186(41.2)	218(48.2)	234(51.8)	311(68.8)	141(31.2)	227(50.2)	225(49.8)	354(78.3)	98(21.7)	430(95.1)	22(4.9)
Hemisphere	*χ*^2^	0.28	*χ*^**2**^	23.34	*χ*^2^	16.1	*χ*^2^	18.51	*χ*^2^	8	*χ*^2^	1.11	*χ*^2^	1.29	*χ*^2^	0.1
	df	1	df	1	df	1	df	1	df	1	df	1	df	1	df	1
	*p*	0.597	*p*	<0.001	*p*	<0.001	*p*	<0.001	*p*	0.004	*p*	0.291	*p*	0.256	*p*	0.752
Country	*c*	47.95	*χ*^2^	70.24	*χ*^2^	125.78	*χ*^2^	98.52	*χ*^2^	54.93	*χ*^2^	31.88	*χ*^2^	39.78	*χ*^2^	41.7
	df	22	df	22	df	22	df	22	df	22	df	22	df	22	df	22
	*p*	0.001	*p*	<0.001	*p*	<0.001	*p*	<0.001	*p*	<0.001	*p*	0.079	*p*	0.012	*p*	0.007

The recommendations derived from the largest number of papers were Management, followed in decreasing order by Policies, General Knowledge, and Conservation in both hemispheres. The Northern Hemisphere presented higher trends and was closer to the total than the Southern Hemisphere, where 136 papers resulted in Management recommendations and fewer than 100 papers resulted in Policies, General Knowledge, and Conservation recommendations (Table 2). In the analysis using the *χ*^2^ test, Table 2 shows that among the papers analysed, no significant differences were detected in the recommendations among countries or between hemispheres.

**Table 2:** Number of observations expressed in absolute frequencies and frequencies relative to the total in percentage (data in parentheses) based on main recommendations derived from socio-ecological studies and their association with the Country and Hemisphere (*χ*^2^ = chi-square test, df = degree of free, *p*-value at <0.005).

	Management of natural systems		Conservation of nature		Policies or governance structures		Scientific-general knowledge	
	No	Yes	No	Yes	No	Yes	No	Yes
*Northern Hemisphere*	50 (11.1)	244 (54)	220 (48.7)	74 (16.4)	135 (29.9)	159 (35.2)	171 (37.8)	123 (27.2)
Norway	0 (0.0)	8 (1.8)	6 (1.3)	2 (0.4)	2 (0.4)	6 (1.3)	8 (1.8)	0 (0.0)
Ireland	2 (0.4)	1 (0.2)	3 (0.7)	0 (0.0)	2 (0.4)	1 (0.2)	1 (0.2)	2 (0.4)
Switzerland	1 (0.2)	2 (0.4)	2 (0.4)	1 (0.2)	0 (0.0)	3 (0.7)	2 (0.4)	1 (0.2)
Germany	4 (0.9)	7 (1.6)	8 (1.8)	3 (0.7)	5 (1.1)	6 (1.3)	6 (1.3)	5 (1.1)
Sweden	1 (0.2)	6 (1.3)	3 (0.7)	4 (0.9)	2 (0.4)	5 (1.1)	5 (1.1)	2 (0.4)
Netherlands	3 (0.7)	3 (0.7)	4 (0.9)	2 (0.4)	4 (0.9)	2 (0.4)	3 (0.7)	3 (0.7)
Finland	1 (0.2)	2 (0.4)	1 (0.2)	2 (0.4)	1 (0.2)	2 (0.4)	1 (0.2)	2 (0.4)
United Kingdom	0 (0.0)	12 (2.7)	10 (2.2)	2 (0.4)	5 (1.1)	7 (1.6)	7 (1.6)	5 (1.1)
Belgium	0 (0.0)	3 (0.7)	2 (0.4)	1 (0.2)	0 (0.0)	3 (0.7)	2 (0.4)	1 (0.2)
Canada	4 (0.9)	20 (4.4)	18 (4.0)	6 (1.3)	14 (3.1)	10 (2.2)	14 (3.1)	10 (2.2)
United States	8 (1.8)	61 (13.5)	56 (12.4)	13 (2.9)	32 (7.1)	37 (8.2)	35 (7.7)	34 (7.5)
Austria	0 (0.0)	3 (0.7)	2 (0.4)	1 (0.2)	2 (0.4)	1 (0.2)	1 (0.2)	2 (0.4)
Japan	5 (1.1)	9 (2.0)	11 (2.4)	3 (0.7)	8 (1.8)	6 (1.3)	9 (2.0)	5 (1.1)
Spain	3 (0.7)	41 (9.1)	35 (7.7)	9 (2.0)	22 (4.9)	22 (4.9)	29 (6.4)	15 (3.3)
France	4 (0.9)	16 (3.5)	11 (2.4)	9 (2.0)	9 (2.0)	11 (2.4)	11 (2.4)	9 (2.0)
Russia	2 (0.4)	9 (2.0)	11 (2.4)	0 (0.0)	7 (1.6)	4 (0.9)	6 (1.3)	5 (1.1)
China	12 (2.7)	41 (9.1)	37 (8.2)	16 (3.5)	20 (4.4)	33 (7.3)	31 (6.9)	22 (4.9)
*Southern Hemisphere*	22 (4.9)	136 (30.1)	122 (27)	36 (8)	59 (13.1)	99 (21.9)	103 (22.8)	55 (12.2)
Australia	9 (2.0)	38 (8.4)	40 (8.9)	7 (1.6)	16 (3.5)	31 (6.9)	23 (5.1)	24 (5.3)
New Zealand	1 (0.2)	15 (3.3)	13 (2.9)	3 (0.7)	7 (1.6)	9 (2.0)	14 (3.1)	2 (0.4)
Chile	5 (1.1)	30 (6.6)	27 (6.0)	8 (1.8)	18 (4.0)	17 (3.8)	29 (6.4)	6 (1.3)
Argentina	1 (0.2)	20 (4.4)	13 (2.9)	8 (1.8)	5 (1.1)	16 (3.5)	13 (2.9)	8 (1.8)
Botswana	2 (0.4)	5 (1.1)	7 (1.6)	0 (0.0)	0 (0.0)	7 (1.6)	5 (1.1)	2 (0.4)
South Africa	4 (0.9)	28 (6.2)	22 (4.9)	10 (2.2)	13 (2.9)	19 (4.2)	19 (4.2)	13 (2.9)
Total	72(15.9)	380(84.1)	342(75.7)	110(24.3)	194(42.9)	258(57.1)	274(60.6)	178(39.4)
Hemisphere	*χ*^2^	0.73	*χ*^2^	0.32	*χ*^2^	3.09	*χ*^2^	2.13
	df	1	df	1	df	1	df	1
	*p*	0.393	*p*	0.573	*p*	0.079	*p*	0.145
Country	*χ*^2^	35.19	*χ*^2^	27.62	*χ*^2^	27.34	*χ*^2^	28.09
	df	22	df	22	df	22	df	22
	*p*	0.037	*p*	0.188	*p*	0.198	*p*	0.173

**Fig. 5:**
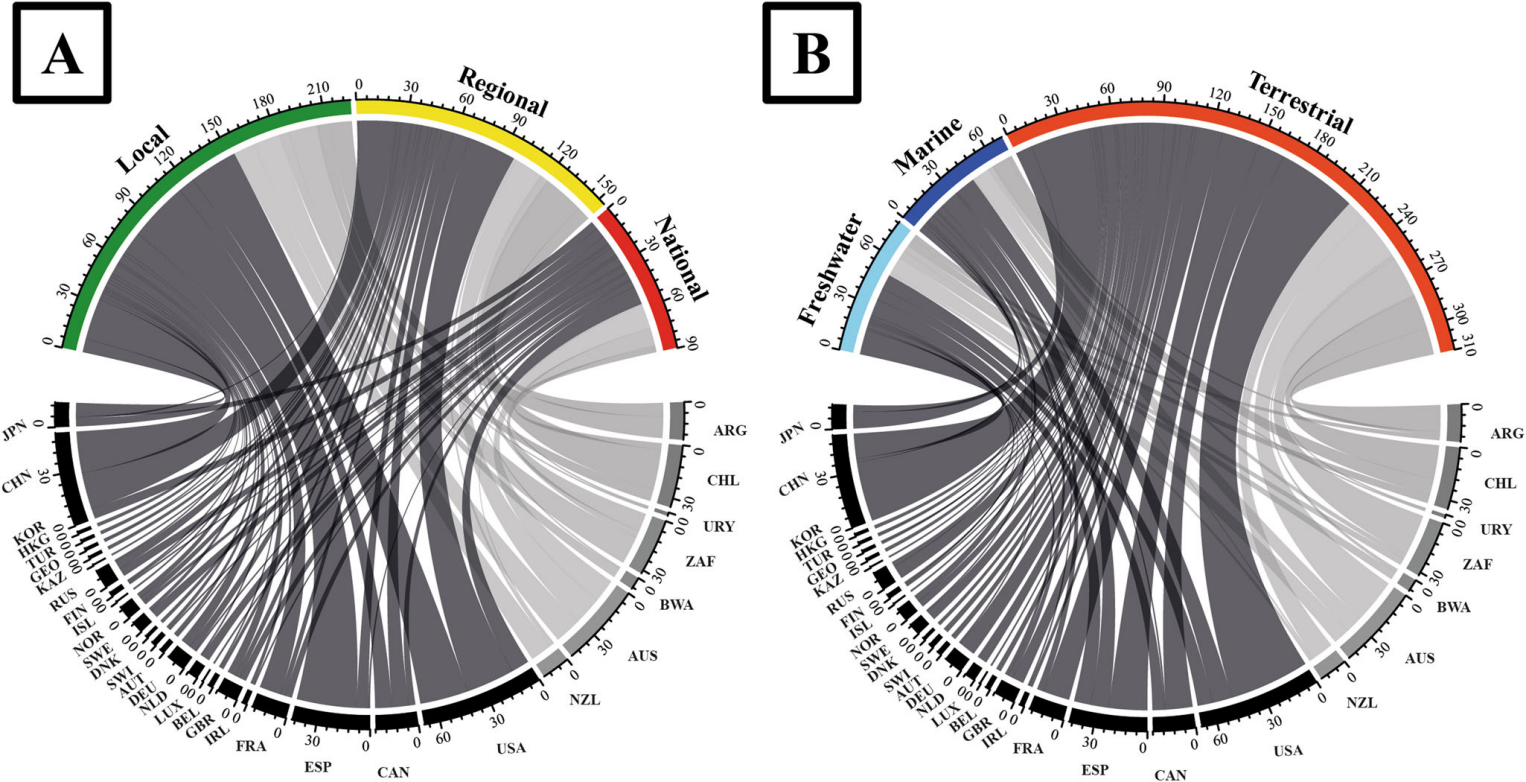
Chord diagrams illustrating the spatial scale proportion and the different prominent natural systems. Spatial scales (**A**) and predominant natural systems (**B**) of the socio-ecological papers (*n* = 467 papers) from 1992 to 2021 (see Annex 1 for countries code).

Most of the studies were at the local level (47%), followed by regional (33%) and national (20%) studies (Fig. 5). In turn, studies in terrestrial systems are the ones that have been carried out the most (69%); more than four times the number of studies carried out in terrestrial systems than in freshwater (16%) and marine systems (15%).

**Fig. 6:**
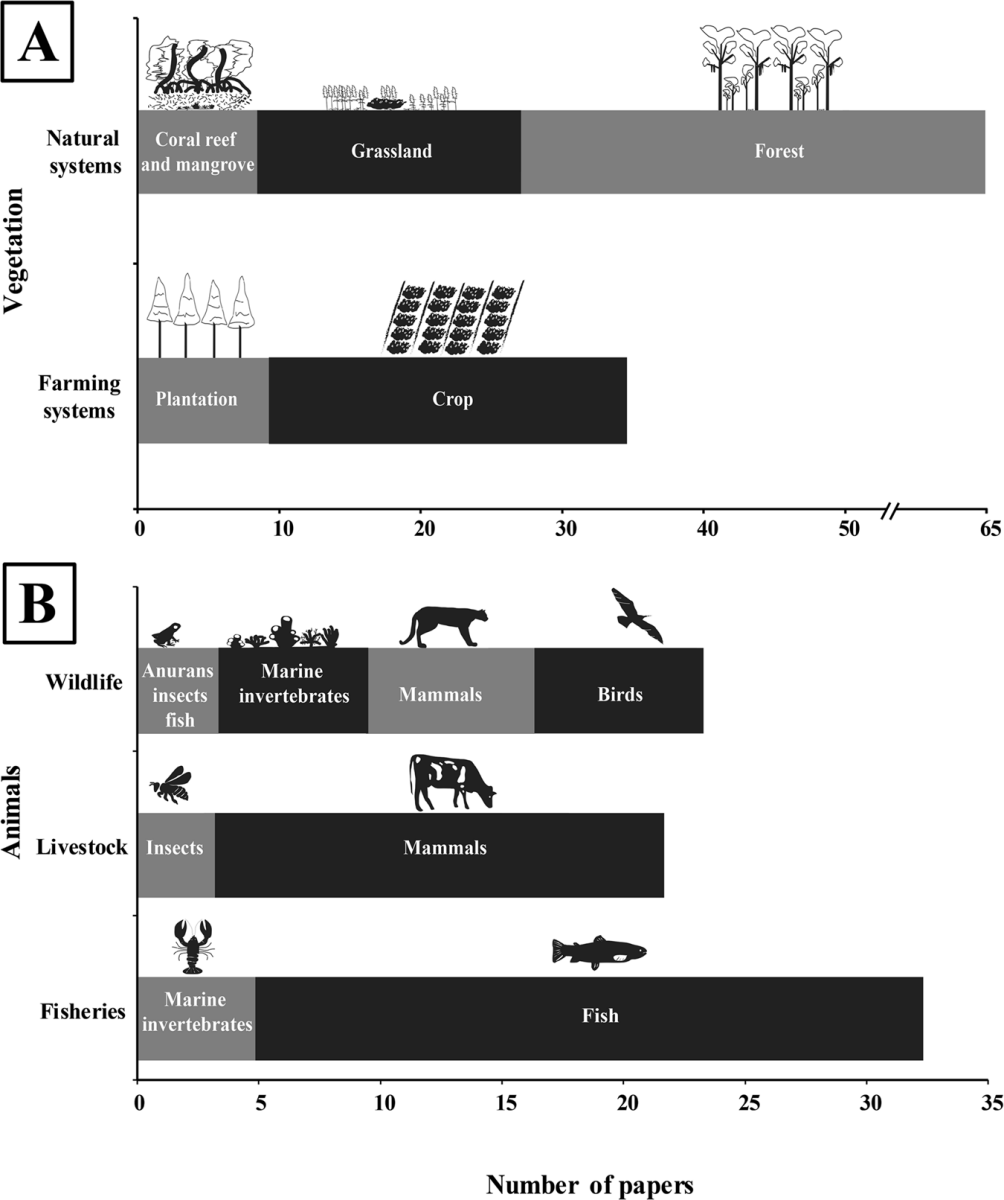
Graphs illustrating the quantity of socio-ecological studies on flora and fauna. Number of socio-ecological papers examining the flora (*n* = 100) (**A**) and fauna (*n* = 79) (**B**) issues in this review.

Among the 467 selected papers, 38% were focused on the relationship between humans and aspects of nature (Fig. 6), whereas most of the studies specifically analysed the relationship between humans and flora (*n* = 100 papers) or fauna (*n* = 79 papers). The highest proportion of the vegetation papers was conducted on natural systems (mainly native forests) (65%), and to a lesser extent, on forest plantations or crops (35%). Regarding the fauna, 70% of the studies were conducted in terrestrial (*n* = 22) and aquatic (*n* = 33) production systems, where livestock (mainly bovine) and aquatic fisheries (e.g., salmon, artisanal coastal fishing, trout) were the most studied groups. Wildlife represented 30% of the studies on animals, where marine invertebrates (e.g., collars), mammals and birds were the most studied groups.

## Discussion

### Socio-ecology by hemisphere in countries with highest HDI and its recommendations

Papers that seek to address the man-nature relationship from a socio-ecological perspective have increased exponentially from 2006 to 2019. However, this trend has decreased since 2019, which should be confirmed in the future (perhaps due to the 2019 COVID pandemic). The socio-ecological approach describes the relationship among various social and environmental aspects of a specific location (e.g., sustainable development aims). In both hemispheres, socio-ecology has evolved in high HDI countries, where it is employed as a normative term and to foster the essential laws and regulations that direct the formation of international agreements concerning social and environmental issues on a global scale (Garmestani et al., 2013; Sabato et al., 2021). However, although socio-ecology faces the challenge of developing new public policies for nature conservation and social welfare (Shackleton et al., 2011; Ruiz-Ballesteros and Gálvez-García, 2014), most of the recommendations are focused on terrestrial management (see Fig. 4, Tables 1 and 2). These recommendations focus are interesting because the oceans are becoming increasingly important. In fact, in 2021, the United Nations declared the Decade of the Oceans (https://en.unesco.org/ocean-decade). Despite the growing importance of ocean protection and conservation globally, the scientific community makes efforts to understand the management of the terrestrial ecosystem, where socio-ecology plays a preponderant role in environmental and social issues in countries such as South Africa (e.g., Le Maitre et al., 2007; Gammage et al., 2019), Argentina (e.g., Ianni and Geneletti, 2010; Laterra et al., 2016), Chile (e.g., De Juan et al., 2015), Spain (e.g., Morán-Ordóñez et al., 2013; Garau et al., 2020), France (e.g., De Chazal et al., 2008; Tufféry et al., 2021), Norway (e.g., Bredin et al., 2015; Johansen et al., 2019), Japan (e.g., Kabaya et al., 2019; Fukamachi, 2020), among others. Natural resources management includes recommendations from a socio-ecological perspective in the countries analysed. However, other recommendations pointed to the generation of public policies (e.g., Arnaiz-Schmitz et al., 2018; Simpson and Bagelman, 2018), the vulnerability or resilience of terrestrial systems (e.g., De Chazal et al., 2008; Domptail and Easdale, 2013; Barnes and Nel, 2017), adaptive management of the natural resources (e.g., De Juan et al., 2015; Berninsone et al., 2018), ethnographic approaches (e.g., Mellado et al., 2019), among others.

Socio-ecology addresses a wide range of problems (Martínez-Fernández et al., 2021). In Japan, after the 2011 Tsunami, socio-ecological studies began to consider disaster areas and their relationship with people and the environment (e.g., Takeuchi et al., 2014). Socio-ecology has also approached topics of research on medicine and public health (e.g., Edwards et al., 2004; Tong, 2004), and in recent years, it has helped formulate management schemes and programmes for social and ecological situations related to the COVID-19 pandemic (e.g., Powers et al., 2021; Nhamo and Ndlela, 2021). Therefore, there is an opportunity to continue diversifying experiences from different countries (Ossola and Hopton, 2018; Pan et al., 2019). Another advantage provided by the socio-ecological approach was the chance to provide a scheme to improve governance relationships in both terrestrial and marine-freshwater systems (Thomsen, 2008; Mee et al., 2016; McKay et al., 2020), which also provides a basis for developing actions that minimize impacts on nature and support the sustainability visions of both the environment and society (De Chazal et al., 2008; Marshall et al., 2013).

### Predominant natural systems and spatial scales at which socio-ecology predominates

In reviewed papers, the authors cover topics at different spatial scales of socio-ecological phenomena that they consider essential in predominant natural systems such as marine, freshwater, and terrestrial systems. However, studies conducted in freshwater and marine systems were fewer than in terrestrial ones, as suggested by Link and Marshak (2019). On the other hand, the scales of analyses are because they are used to improve the management, policies, or knowledge based on national (e.g., countries), regional (e.g., provinces) or local (e.g., basins) entities that are beneficial for the environment and people (Ekbia and Evans, 2009; Leys and Vanclay, 2011; Bode et al., 2016). For example, community-based research on local socio-ecological systems provides a framework for public participation in natural resource management (Thomsen, 2008; Morán-Ordóñez et al., 2013; Rasch et al., 2017) and food security (Pereira and Ruysenaar, 2012). However, complex interactions within socio-ecological systems are difficult to empirically put into practice in environmental assessments (Shackleton et al., 2011; Nair et al., 2016). Socio-ecological systems are seldom closed or static (Brewer, 2012; Drewes and Silbernagel, 2012), so it is interesting to know how sociological studies are located at a local scale (e.g., watersheds). This scale of social relationships typically generates better proposals for connecting local economies with natural assets such as the use of water resources (Ruiz-Ballesteros and Gálvez-García, 2014; Conrad and Yates, 2018; Mistry et al., 2021). For instance, river basins increasingly serve as organizing units to assess and manage human impacts on the environment (Mayer et al., 2014). Socio-ecological studies at regional and national, and even international scales, can be explained by the need to understand environmental risks and climate change (e.g., Marshall et al., 2012; Ford et al., 2013; Williams et al., 2020) or prioritizing protected wild areas (e.g., De Aranzabal et al., 2008; Ianni and Geneletti, 2010; Mathevet et al., 2016).

### Aspects of nature concerning socio-ecological studies

The socio-ecological studies show different values for aspects of nature concerning biodiversity in both hemispheres. These values reveal aspects of nature where the importance of understanding social attitudes toward species management strategies (e.g., both for native and exotic species) and their social perceptions are emphasised (e.g., Lewis et al., 2019; Jung, 2020; Huertas Herrera et al., 2021). The sustainable conservation and management of ecosystem services are based on understanding the socio-ecological system dynamics (Drewes and Silbernagel, 2012; Barton et al., 2020), where forests play a leading role (e.g., Olander et al., 2021). Forest landscapes are typical socio-ecological systems shaped by forest management (Fortin et al., 2019; Tufféry et al., 2021). Less than half of the papers analysed, especially considering biodiversity (flora and fauna), denote a dissociation of most studies with nature. In cases where the papers were related to biodiversity, several socio-ecological studies are related to conspicuous species of mammals (e.g., Davis et al., 2020; Jung, 2020) or birds (e.g., Celis-Diez et al., 2017), and to a lesser extent, reptiles, or amphibians (e.g., Jellinek et al., 2014).

However, studies have been done in these groups, revealing efforts to, for example, understand problems related to wildlife, for example, indicator species (Domroese and Johnson, 2017). Part of this can be explained because many socio-ecological conflicts occur between carnivores and producers (e.g., Gáspero et al., 2018; Expósito-Granados et al., 2019; Bonamy et al., 2020). On the other hand, birds are an emblematic animal group for managing and conserving natural areas, with numerous initiatives related to tourism (De Aranzabal et al., 2008; Solstrand, 2013; Outeiro et al., 2018). It is worth mentioning how insects have been part of socio-ecological studies regarding domestic fauna. These studies can be explained by the social and environmental potential of honey production from bees (e.g., Giacobino et al., 2018). On the other hand, the number of socio-ecological studies related to livestock reveals the interest of the scientific community in solving problems related to environmental conflicts derived from the management of farms (e.g., Pereira and Ruysenaar, 2012; Lamarque et al., 2013; Shapero et al., 2018; Barton et al., 2020). Like livestock, agricultural practices are communally related to social problems, seeking to solve food security problems (Lamarque et al., 2013; Spiegelaar et al., 2013; Rasch et al., 2016; Shilomboleni and De Plaen, 2019). Land-use changes are frequently triggered by incentives (e.g., reforestation or crops), causing tensions in rural communities (e.g., Leys and Vanclay, 2011; Pereira, 2013; Jellinek et al., 2014; Rasch et al., 2017).

Fisheries were one of the most interesting socio-ecological aspects in countries from both hemispheres. The high number of papers studying fisheries can be explained by the high demand for coastal natural resources, for example, artisanal fishing (Sowman, 2011; Mee et al., 2016; Berninsone et al., 2018), which requires resource managers to implement policies that are increasingly restrictive for resource users (Holt et al., 2012; Marshall and Marshall, 2007). In the Southern Hemisphere, in countries such as Australia, socio-ecological studies focus on the conservation of coral reefs, and this can explain the importance of such sensitive and attractive ecosystems for people (e.g., Marshall et al., 2013; Bode et al., 2016; Goldberg et al., 2018). Therefore, many studies were less concerned with nature conservation, but with so many social problems to be solved, even in the most developed countries, it seems not a priority when natural system management and the generation of public policies are needed.

The social and environmental history can explain these ecological differences between several northern hemisphere countries and those in the southern hemisphere, which may be, for example, a reflection of European’s political hegemony during the 19th century’s globalization and the subsequent division of the world between the West and the East (Berger, 2004). Power disparities between developed and developing countries are highlighted by concepts like “global south” and “global north” (Demeny, 1981; Armillas-Tiseyra and Mahler, 2022). These ideas apply to socioeconomic positions rather than specific geographical areas. For instance, although being in the Southern Hemisphere, Australia and New Zealand are regarded as a nation of the “global north” because of their advanced economy (Shariff, 1997).

There are widely used indices that rank countries based on purely economic indicators. For example, indices that rank countries from the least free to the freest (e.g., Index of Economic Freedom) through scores based on the economic freedom of each country (Gwartney et al., 2021; Miller et al., 2022). However, these indices do not include freedom in a socially broad sense, as they focus more on four broad policy areas that affect economic freedom (high-income economies): the rule of law, government size, regulatory efficiency, and open markets (Miller et al., 2022). In this context, the HDI considers a social and environmental development concept closer to a socio-ecological notion. The HDI can be used to challenge national policy decisions, such as how two countries with the same gross national income per capita can have such disparities in human development outcomes (UNDP, 2020b). This could serve to understand some of these countries’ social and environmental prospects.

Despite all countries showing a comparable HDI, there is a hemispheric difference in socio-ecological studies. This might be because scientific research is disturbingly unequal (Czerniewicz, 2015), being research in the Northern Hemisphere more prominent than in the Southern Hemisphere. This is related to the higher capacity of Northern countries to conduct research (Blicharska et al., 2017). A possible explanation may be that scientific capabilities are different due to the budgets allocated to doing research (Richards, 2022). Actually, in addition to working harder, scientists in emerging economies who want to catch up with the developed world must look for a variety of financial alternatives due to publication expenses (Vuong, 2018; Richards, 2022). In this context, Nguyen and Vuong (2021) showed international cooperation is a successful strategy for raising scientific quality and quantity on global agendas (e.g., Aichi Biodiversity Targets), due to the greater possibilities of obtaining financing. Also, perhaps, due to the availability of connections to institutions or researchers who developed the theories and methods that underpin these investigations (Karlsson et al., 2007). Thus, even if they all have high HDIs, northern hemisphere nations have greater funding for scientific research. For instance, Blicharska et al. (2017) found that most authors of articles discussing climate change are from high-income economies (such as those in the OECD), with only a tiny fraction originating from Southern countries. Continued human population growth and increasing consumption have resulted in unsustainable exploitation of biological diversity, aggravated by climate change and other anthropogenic environmental impacts (Rands et al., 2010). The situation that the whole world is going through around climate change makes many nations think in socio-ecological terms (Williams et al., 2020; Birkmann et al., 2022). One of the notable aspects is that we live at a turning point that invites us to think about social, institutional, and environmental relations to take on the challenges related to climate change (Ivanova, 2020). To do this, efforts in science must enhance international collaboration between the northern and southern hemispheres because climate change must be addressed at different scales, national and international (Ford et al., 2013; Lamarque et al., 2013; Blicharska et al., 2017).

The power of adopting scientific knowledge is asymmetrical because it is not the same in social and economic terms if it comes from certain developed nations as opposed to developing ones (Merton, 1968). It could be expected that the socio-ecological studies derived from high HDI countries tend to participate more strongly in constructing the international order. Lower HDI countries need to meet more basic needs than high HDI countries, which places them in a framework of vulnerability to generate their knowledge. This can explain, among other things, why one hemisphere focuses more on terrestrial systems rather than aquatic or coastal and how they address the conservation challenges of production systems, which are the focus of international agendas and commitments to reduce carbon emissions into the atmosphere.

### Limitations

Transparency allows the public to understand how science works when there are grey areas of disagreement (Vuong, 2020). The main limitations of this study can be: (i) we used the first author´s affiliation contrasted with the hemisphere location of the country where studies were developed to explore the knowledge exchange between both hemispheres. For example, we considered a paper carried out in South Africa in which the first author’s affiliation was an institution based in the United States as a knowledge exchange from the Northern to the Southern Hemisphere. (ii) the fact that our review article is a quantitative survey of the literature on socio-ecological studies is one of the paper’s weaknesses in terms of data and methodology. (iii) SCImago and Scopus may not be unbiased data sources, and some have questioned whether the list of papers indexed by these systems accurately reflects worldwide scientific production (Tennant, 2020). Indeed, this data source has recognized stronger limitations in social sciences, for which other datasets are usually recommended (e.g., Web of Science, Latindex). We could suggest that the scope of SCImago limits our data collection technique and we declare this a limitation of this study. However, this dataset was considered wide enough to extract consistent trends, it is constantly updated, and is available in many institutions. Of course, no methodology is perfect; they all have advantages and disadvantages, but we do not believe this “possible weakness” has a significant impact on the findings, and we believe this provides a good picture of the socio-ecological-based articles across the two hemispheres, as well as how the scale, scope, and approach differ among the high HDI valued countries. For example, this study can be relevant for: (i) Socio-ecological policies towards biodiversity can be risen for sustainable ecosystem management purposes promoting collaborative research and reducing the friction in international collaboration. (ii) Through the Scopus and HDI data, it is possible to show some likely connections that academic journals have in the Southern and Northern Hemispheres to identify socio-ecological patterns that may be of interest in human relationships with natural systems.

## Concluding remarks

The information presented here refers to countries with a high HDI, which reveals how socio-ecological studies are used to solve issues of marine, freshwater, and terrestrial systems. We depicted that the leading countries in these studies differ in the Northern and Southern hemispheres. In particular, the main exchange of socio-ecological knowledge occurs from North America and Europe to the Southern Hemisphere. The transfer seems to result from the proportion of socio-ecological publications in each hemisphere (mainly the Northern Hemisphere). Overall, the studies in terrestrial systems are the ones that have been carried out the most compared to freshwater and marine systems. Our analysis shows that studies focused on socio-ecology have increased in recent years (since 2006), where most socio-ecological studies generated management recommendations, followed by policy recommendations in both hemispheres. However, these subject areas and recommendations come mainly from studies in the northern hemisphere with the largest number of papers. The relative importance of these subject areas was broadly used across multiple spatial scales. Such scales in socio-ecological studies provide opportunities to engage people with flora and fauna, where native forests generated better social spaces to improve the sustainable management of the natural systems than forest plantations or crops. The reviewed studies highlight socio-ecological critical research on productive systems such as livestock and marine fisheries. Based on the results, the socio-ecology approach was used in the analysed countries with greater social and economic development indicators to develop management options for the natural systems.

## Supplementary information

Supplementary information

## Data Availability

All available data and materials are included in the manuscript.

## References

[CR1] Angrist N, Djankov S, Goldberg PK, Patrinos HA (2021). Measuring human capital using global learning data. Nature.

[CR2] Armillas-Tiseyra M, Mahler AG (2022). Introduction: new critical directions in global south studies, continuing the conversation. Comp Lit Stud.

[CR3] Arnaiz-Schmitz C, Schmitz M, Herrero-Jáuregui C, Gutiérrez-Angonese J, Pineda F, Montes C (2018). Identifying socio-ecological networks in rural–urban gradients: diagnosis of a changing cultural landscape. Sci Total Environ.

[CR4] Balland P-A, Jara-Figueroa C, Petralia SG, Steijn MPA, Rigby DL, Hidalgo CA (2020). Complex economic activities concentrate in large cities. Nat Hum Behav.

[CR5] Barnes A, Nel V (2017). Putting spatial resilience into practice. Urban Forum.

[CR6] Barton E, Bennett DE, Burnidge W (2020). Holistic perspectives: Understanding rancher experiences with holistic resource management to bridge the gap between rancher and researcher perspectives. Rangelands.

[CR7] Benjamin DJ, Berger JO, Johannesson M, Nosek BA, Wagenmakers E-J, Berk R, Bollen KA, Brembs B, Brown L, Camerer C, Cesarini D, Chambers CD, Clyde M, Cook TD, De Boeck P, Dienes Z, Dreber A, Easwaran K, Efferson C, Fehr E, Fidler F, Field AP, Forster M, George EI, Gonzalez R, Goodman S, Green E, Green DP, Greenwald AG, Hadfield JD, Hedges, Larry V, Held L, Hua Ho T, Hoijtink H, Hruschka DJ, Imai K, Imbens G, Ioannidis JPA, Jeon M, Jones JH, Kirchler M, Laibson D, List J, Little R, Lupia A, Machery E, Maxwell SE, McCarthy M, Moore DA, Morgan SL, Munafó M, Nakagawa S, Nyhan B, Parker TH, Pericchi L, Perugini M, Rouder J, Rousseau J, Savalei V, Schönbrodt FD, Sellke T, Sinclair B, Tingley D, Van Zandt T, Vazire S, Watts DJ, Winship C, Wolpert RL, Xie Y, Young C, Zinman J, Johnson VE (2018). Redefine statistical significance. Nat Hum Behav.

[CR8] Berger MT (2004). After the Third World? History, destiny and the fate of Third Worldism. Third World Q.

[CR9] Berninsone LG, Newton A, Icely J (2018). A co-designed, transdisciplinary adaptive management framework for artisanal fisheries of Pehuen Co and Monte Hermoso (Argentina). Ocean Coast Manag.

[CR10] Birkmann J, Jamshed A, McMillan JM, Feldmeyer D, Totin E, Solecki W, Ibrahim ZZ, Roberts D, Kerr RB, Poertner H-O, Pelling M, Djalante R, Garschagen M, Leal Filho W, Guha-Sapir D, Alegría A (2022). Understanding human vulnerability to climate change: a global perspective on index validation for adaptation planning. Sci Total Environ.

[CR11] Blicharska M, Smithers RJ, Kuchler M, Agrawal GK, Gutiérrez JM, Hassanali A, Huq S, Koller SH, Marjit S, Mshinda HM, Masjuki HH, Solomons NW, Van Staden J, Mikusiński G (2017). Steps to overcome the North–South divide in research relevant to climate-change policy and practice. Nat Clim Change.

[CR12] Bode M, Sanchirico JN, Armsworth PR (2016). Returns from matching management resolution to ecological variation in a coral reef fishery. Proc R Soc B.

[CR13] Bonamy M, Herrmann TM, Harbicht AB (2020). ‘I think it is the toughest animal in the North’: human-wolverine interactions among hunters and trappers in the Canadian Northwest Territories. Polar Geogr.

[CR14] Bredin YK, Lindhjem H, van Dijk J, Linnell JD (2015). Mapping value plurality towards ecosystem services in the case of Norwegian wildlife management: AQ analysis. Ecol Econ.

[CR15] Brewer JF (2012). Don’t fence me in: boundaries, policy, and deliberation in Maine’s lobster commons. Ann Assoc Am Geogr.

[CR16] Celis-Diez JL, Muñoz CE, Abades S, Marquet PA, Armesto JJ (2017). Biocultural homogenisation in urban settings: public knowledge of birds in city parks of Santiago, Chile. Sustainability.

[CR17] Colding J, Barthel S (2019). Exploring the social–ecological systems discourse 20 years later. Ecol Soc.

[CR18] Conrad S, Yates D (2018). Coupling stated preferences with a hydrological water resource model to inform water policies for residential areas in the Okanagan Basin, Canada. J Hydrol.

[CR19] Czerniewicz L (2015) This map of the world’s scientific research is disturbingly unequal. https://qz.com/449405/this-map-of-the-worlds-scientific-research-is-disturbingly-unequal/. Accessed 25 Jun 2022

[CR20] Davis KP, Augustine DJ, Monroe AP, Derner JD, Aldridge CL (2020). Adaptive rangeland management benefits grassland birds utilising opposing vegetation structure in the shortgrass steppe. Ecol Appl.

[CR21] De Aranzabal I, Schmitz M, Aguilera P, Pineda F (2008). Recreation suitability analysis: Application in protected and non-protected areas. WIT Trans Ecol Environ.

[CR22] De Chazal J, Quétier F, Lavorel S, Van Doorn A (2008). Including multiple differing stakeholder values into vulnerability assessments of socio-ecological systems. Glob Environ Change.

[CR23] De la Escosura LP (2015). World human development: 1870–2007. Rev Income Wealth.

[CR24] De la Escosura LP (2021). Augmented human development in the age of globalization. Econ Hist Rev.

[CR25] De Juan S, Gelcich S, Ospina-Alvarez A, Perez-Matus A, Fernandez M (2015). Applying an ecosystem service approach to unravel links between ecosystems and society in the coast of central Chile. Sci Total Environ.

[CR26] Demeny P (1981). The North–South income gap: a demographic perspective. Popul Dev Rev.

[CR27] De Vos A, Reinette B, Preiser R (2019). Methods for understanding social-ecological systems: a review of place-based studies. Ecol Soc.

[CR28] Domptail S, Easdale MH (2013). Managing socio‐ecological systems to achieve sustainability: a study of resilience and robustness. Environ Policy Gov.

[CR29] Domroese MC, Johnson EA (2017). Why watch bees? Motivations of citizen science volunteers in the Great Pollinator Project. Biol Conserv.

[CR30] Drewes AD, Silbernagel J (2012). Uncovering the spatial dynamics of wild rice lakes, harvesters and management across Great Lakes landscapes for shared regional conservation. Ecol Modell.

[CR31] Edwards N, Mill J, Kothari AR (2004). Multiple intervention research programs in community health. Can J Nurs Res.

[CR32] Ekbia HR, Evans TP (2009). Regimes of information: Land use, management, and policy. Inf Soc.

[CR33] Expósito-Granados M, Castro AJ, Lozano J, Aznar-Sanchez JA, Carter NH, Requena-Mullor JM, Malo AF, Olszańska A, Morales-Reyes Z, Moleón M (2019). Human–carnivore relations: conflicts, tolerance and coexistence in the American West. Environ Res Lett.

[CR34] Ford JD, McDowell G, Shirley J, Pitre M, Siewierski R, Gough W, Duerden F, Pearce T, Adams P, Statham S (2013). The dynamic multiscale nature of climate change vulnerability: an Inuit harvesting example. Ann Assoc Am Geogr.

[CR35] Fortin M, Pichancourt J-B, de Melo LC, Colin A, Caurla S (2019). The effect of stumpage prices on large-area forest growth forecasts based on socio-ecological models. Forestry.

[CR36] Fukamachi K (2020). Building resilient socio-ecological systems in Japan: Satoyama examples from Shiga Prefecture. Ecosyst Serv.

[CR37] Furlan M, Mariano E (2021). Guiding the nations through fair low-carbon economy cycles: a climate justice index proposal. Ecol Indic.

[CR38] Gammage LC, Jarre A, Mather C (2019). A changing fishery system: perspectives from crew in the Southern Cape’s handline fishery. S Afr Geogr J.

[CR39] Garau E, Vila-Subiros J, Pueyo-Ros J, Ribas Palom A (2020). Where do ecosystem services come from? assessing and mapping stakeholder perceptions on water ecosystem services in the Muga River Basin (Catalonia, Spain). Land.

[CR40] Garmestani AS, Allen CR, Benson MH (2013). Can Law foster social–ecological resilience?. Ecol Soc.

[CR41] Gáspero PG, Easdale MH, Pereira JA, Fernández-Arhex V, Von Thüngen J (2018). Human-carnivore interaction in a context of socio-productive crisis: assessing smallholder strategies for reducing predation in North-west Patagonia, Argentina. J Arid Environ.

[CR42] Giacobino A, Pacini A, Molineri A, Rodríguez G, Crisanti P, Bulacio Cagnolo N, Merke J, Orellano E, Bertozzi E, Pietronave H, Signorini M (2018). Potential associations between the mite Varroa destructor and other stressors in honeybee colonies (*Apis mellifera* L.) in temperate and subtropical climate from Argentina. Prev Vet Med.

[CR43] Goddard H (1969). Hemispheres North and South. Economic disparity among nations. Hisp Am Hist Rev.

[CR44] Goldberg J, Birtles A, Marshall N, Curnock M, Case P, Beeden R (2018). The role of Great Barrier Reef tourism operators in addressing climate change through strategic communication and direct action. J Sustain Tour.

[CR45] Gu Z, Gu L, Eils R, Schlesner M, Brors B (2014). Circlize implements and enhances circular visualisation in R. Bioinform.

[CR46] Gwartney J, Lawson R, Hall J, Murphy R (2021) Economic freedom of the world: 2021 annual report. Fraser Institute

[CR47] Holt TV, Moreno CA, Binford MW, Portier KM, Mulsow S, Frazer TK (2012). Influence of landscape change on nearshore fisheries in southern Chile. Glob Change Biol.

[CR48] Huertas Herrera A, Toro Manríquez M, Lencinas MV, Martínez Pastur G (2021) The North American Beaver Invasion and the Impact Over the Ecosystem Services in the Tierra del Fuego Archipelago. In: Peri PL, Martínez Pastur G, Nahuelhual L (eds) Ecosystem services in Patagonia: a multi-criteria approach for an integrated assessment. Springer, Cham, pp. 213–226

[CR49] Ianni E, Geneletti D (2010). Applying the ecosystem approach to select priority areas for forest landscape restoration in the Yungas, Northwestern Argentina. Environ Manage.

[CR50] Ivanova M (2020). Everyone, everywhere: the global challenge of climate change. Nature.

[CR51] Jellinek S, Rumpff L, Driscoll DA, Parris KM, Wintle BA (2014). Modelling the benefits of habitat restoration in socio-ecological systems. Biol Conserv.

[CR52] Johansen L, Taugourdeau S, Hovstad KA, When S (2019). Ceased grazing management changes the ecosystem services of semi-natural grasslands. Ecosyst People.

[CR53] Jung TS (2020). Investigating local concerns regarding large mammal restoration: Group size in a growing population of reintroduced bison (*Bison bison*). Glob Ecol Conserv.

[CR54] Kabaya K, Hashimoto S, Fukuyo N, Uetake T, Takeuchi K (2019). Investigating future ecosystem services through participatory scenario building and spatial ecological-economic modelling. Sustain Sci.

[CR55] King N, Biggs H, Loon R (2007). Seeking common ground: how natural and social scientists might jointly create an overlapping worldview for sustainable livelihoods: a South African perspective. Conserv Soc.

[CR56] Kowalski AM (2020) Global South-Global North Differences. In: Leal Filho W, Azul A, Brandli L, Lange Salvia A, Özuyar P, Wall T (eds) No poverty. Encyclopedia of the UN sustainable development goals. Springer, Cham, pp. 1–12

[CR57] Lamarque P, Artaux A, Barnaud C, Dobremez L, Nettier B, Lavorel S (2013). Taking into account farmers’ decision making to map fine-scale land management adaptation to climate and socio-economic scenarios. Landsc Urban Plan.

[CR58] Laterra P, Barral P, Carmona A, Nahuelhual L (2016). Focusing conservation efforts on ecosystem service supply may increase vulnerability of socio-ecological systems. PLoS ONE.

[CR59] Lau J (2006) The impact of information competencies on socio-economic development in Southern Hemisphere economies. In: Martin A, Madigan D (eds) Digital literacies for learning. UK, p. 152

[CR60] Jha S, McCawley P (2011) South–South economic linkages: an overview. Asian Development Bank Economics Working Paper (270), Philippines

[CR61] Karlsson S, Srebotnjak T, Gonzales P (2007). Understanding the North–South knowledge divide and its implications for policy: a quantitative analysis of the generation of scientific knowledge in the environmental sciences. Environ Sci Policy.

[CR62] Le Maitre DC, Milton SJ, Jarmain C, Colvin CA, Saayman I, Vlok JH (2007). Linking ecosystem services and water resources: landscape‐scale hydrology of the Little Karoo. Front Ecol Environ.

[CR63] Lewis CL, Granek EF, Nielsen-Pincus M (2019). Assessing local attitudes and perceptions of non-native species to inform management of novel ecosystems. Biol Invasions.

[CR64] Leys AJ, Vanclay JK (2011). Stakeholder engagement in social learning to resolve controversies over land-use change to plantation forestry. Reg Environ.

[CR65] Link JS, Marshak AR (2019). Characterising and comparing marine fisheries ecosystems in the United States: determinants of success in moving toward ecosystem-based fisheries management. Rev Fish Biol Fish.

[CR66] Marshall NA, Marshall PA (2007). Conceptualising and operationalising social resilience within commercial fisheries in northern Australia. Ecol Soc.

[CR67] Marshall NA, Park S, Adger W, Brown K, Howden S (2012). Transformational capacity and the influence of place and identity. Environ Res Lett.

[CR68] Marshall NA, Tobin RC, Marshall PA, Gooch M, Hobday AJ (2013). Social vulnerability of marine resource users to extreme weather events. Ecosystems.

[CR69] Martínez-Fernández J, Banos-González I, Esteve-Selma MÁ (2021). An integral approach to address socio-ecological systems sustainability and their uncertainties. Sci Total Environ.

[CR70] Mathevet R, Thompson JD, Folke C, Chapin FS (2016). Protected areas and their surrounding territory: socioecological systems in the context of ecological solidarity. Ecol Appl.

[CR71] Mayer A, Winkler R, Fry L (2014). Classification of watersheds into integrated social and biophysical indicators with clustering analysis. Ecol Indic.

[CR72] McKay PA, Olabisi LS, Vogt CA (2020). Assessing improvements in socio-ecological system governance using mixed methods and the quality governance framework and its diagnostic capacity tool. Environ Syst Decis.

[CR73] Mee JA, Post JR, Ward H, Wilson KL, Newton E, Cantin A (2016). Interaction of ecological and angler processes: experimental stocking in an open access, spatially structured fishery. Ecol Appl.

[CR74] Mellado MA, Blanco-Wells G, Nahuelhual L, Saavedra G (2019). Livelihood trajectories in the Chilean Patagonian region: an ethnographic approach to coastal and marine socioecological change. Reg Environ Change.

[CR75] Merton RK (1968). The Matthew Effect in science. Science.

[CR76] Miller T, Kim AB, Roberts JM, Tyrrell P (2022) 2022 Index of economic freedom. The Heritage Foundation, Washington, DC

[CR77] Mistry I, Beaudoin C, Kotecha J, Evans H, Stevens M, Vermaire JC, Cooke SJ, Young N (2021). Action research to improve water quality in Canada’s Rideau Canal: how do local groups reshape environmental governance?. Local Environ.

[CR78] Morán-Ordóñez A, Bugter R, Suárez-Seoane S, de Luis E, Calvo L (2013). Temporal changes in socio-ecological systems and their impact on ecosystem services at different governance scales: a case study of heathlands. Ecosystems.

[CR79] Morzillo AT, Colocousis CR, Munroe DK, Bell KP, Martinuzzi S, Van Berkel DB, Lechowicz MJ, Rayfield B, McGill B (2015). Communities in the middle: interactions between drivers of change and place-based characteristics in rural forest-based communities. J Rural Stud.

[CR80] Nair SS, Preston BL, King AW, Mei R (2016). Using landscape typologies to model socioecological systems: application to agriculture of the United States Gulf Coast. Environ Model Softw.

[CR81] Nguyen M-H, Vuong Q-H (2021). Evaluation of the Aichi Biodiversity Targets: the international collaboration trilemma in interdisciplinary research. Pac Conserv Biol.

[CR82] Nhamo L, Ndlela B (2021). Nexus planning as a pathway towards sustainable environmental and human health post Covid-19. Environ Res.

[CR83] Norgaard RB, Kallis G, Kiparsky M (2009). Collectively engaging complex socio-ecological systems: re-envisioning science, governance, and the California Delta. Environ Sci Policy.

[CR84] Olander L, Warnell K, Warziniack T, Ghali Z, Miller C, Neelan C (2021). Exploring the use of ecosystem services conceptual models to account for the benefits of public lands: an example from national forest planning in the United States. Forests.

[CR85] Orach K, Schlüter M (2016). Uncovering the political dimension of social-ecological systems: contributions from policy process frameworks. Glob Environ Change.

[CR86] Ossola A, Hopton ME (2018). Climate differentiates forest structure across a residential macrosystem. Sci Total Environ.

[CR87] Ostrom E (2009). A general framework for analysing sustainability of social–ecological systems. Science.

[CR88] Outeiro L, Villasante S, Oyarzo H (2018). The interplay between fish farming and nature-based recreation-tourism in Southern Chile: a perception approach. Ecosyst Serv.

[CR89] Palla G, Tibély G, Mones E, Pollner P, Vicsek T (2015). Hierarchical networks of scientific journals. Palgrave Commun.

[CR90] Pan H, Zhang L, Cong C, Deal B, Wang Y (2019). A dynamic and spatially explicit modeling approach to identify the ecosystem service implications of complex urban systems interactions. Ecol Indic.

[CR91] Paveglio TB, Prato T, Hardy M (2013). Simulating effects of land use policies on extent of the wildland urban interface and wildfire risk in Flathead County, Montana. J Environ Manage.

[CR92] Pereira LM (2013). The future of the food system: cases involving the private sector in South Africa. Sustainability.

[CR93] Pereira LM, Ruysenaar S (2012). Moving from traditional government to new adaptive governance: the changing face of food security responses in South Africa. Food Secur.

[CR94] Powers M, Brown P, Poudrier G, Ohayon JL, Cordner A, Alder C, Atlas MG (2021). COVID-19 as eco-pandemic injustice: opportunities for collective and antiracist approaches to environmental health. J Health Soc Behav.

[CR95] Pye S, Bradley S, Hughes N, Price J, Welsby D, Ekins P (2020). An equitable redistribution of unburnable carbon. Nat Commun.

[CR96] Rands MR, Adams WM, Bennun L, Butchart SH, Clements A, Coomes D, Entwistle A, Hodge I, Kapos V, Scharlemann JPW, Sutherland WJ, Vira B (2010). Biodiversity conservation: challenges beyond 2010. Science.

[CR97] Rasch S, Heckelei T, Oomen RJ (2016). Reorganising resource use in a communal livestock production socio-ecological system in South Africa. Land Use Policy.

[CR98] Rasch S, Heckelei T, Storm H, Oomen R, Naumann C (2017). Multi-scale resilience of a communal rangeland system in South Africa. Ecol Econ.

[CR99] Richards G (2022) 3 ways to address the North–South divide in scientific research. World Economic Forum. https://europeansting.com/2022/02/07/3-ways-to-address-the-north-south-divide-in-scientific-research/. Accessed 25 June 2022

[CR100] Ruiz-Ballesteros E, Gálvez-García C (2014). Community, common-pool resources and socio-ecological systems: water management and community building in southern Spain. Hum Ecol.

[CR101] Russo S, Sillmann J, Sippel S, Barcikowska MJ, Ghisetti C, Smid M, O’Neill B (2019). Half a degree and rapid socioeconomic development matter for heatwave risk. Nat Commun.

[CR102] Sabato S, Mandelli M, Vanhercke B (2021) The socio-ecological dimension of the EU recovery. From the European Green deal to the recovery and resilience facility. EUROsociAL Programme, Madrid, EUROsociAL Collection No. 24, 63pp

[CR103] Sala JE, Torchio G (2019). Moving towards public policy-ready science: philosophical insights on the social-ecological systems perspective for conservation science. Ecosyst People.

[CR104] Shackleton CM, Shackleton SE, Ellery F, Gambiz J, Scholes BJ, Vogel C, Wynberg R, Abrahamse T (2011). The next decade of environmental science in South Africa: a horizon scan. S Afr Geogr J.

[CR105] Shapero M, Huntsinger L, Becchetti T, Mashiri F, James J (2018). Land manager perceptions of opportunities and constraints of using livestock to manage invasive plants. Rangel Ecol Manag.

[CR106] Shariff I (1997). The north–south divide in an emerging new world economic order. World Aff J Int Issues.

[CR107] Shilomboleni H, De Plaen R (2019). Scaling up research-for-development innovations in food and agricultural systems. Dev Pract.

[CR108] Simpson M, Bagelman J (2018). Decolonising urban political ecologies: the production of nature in settler colonial cities. Ann Assoc Am Geogr.

[CR109] Solstrand MV (2013). Marine angling tourism in Norway and Iceland: finding balance in management policy for sustainability. Nat Resour Forum.

[CR110] Sowman M (2011). New perspectives in small-scale fisheries management: challenges and prospects for implementation in South Africa. Afr J Mar Sci.

[CR111] Spiegelaar NF, Tsuji LJ, Oelbermann M (2013). The potential use of agroforestry community gardens as a sustainable import-substitution strategy for enhancing food security in subarctic Ontario, Canada. Sustainability.

[CR112] Takeuchi K, Elmqvist T, Hatakeyama M, Kauffman J, Turner N, Zhou D (2014). Using sustainability science to analyse social–ecological restoration in NE Japan after the great earthquake and tsunami of 2011. Sustain Sci.

[CR113] Tennant J (2020). Web of Science and Scopus are not global databases of knowledge. Eur Sci Ed.

[CR114] Thomsen DC (2008). Community-based research: facilitating sustainability learning. Australas J Environ Manag.

[CR115] Tong S (2004). Ross River virus disease in Australia: epidemiology, socio-ecology and public health response. Intern Med J.

[CR116] Tufféry L, Davi H, López-García N, Rigolot E, Jean F, Stenger A, Lefévre F (2021). Adaptive measures for mountain Mediterranean forest ecosystem services under climate and land cover change in the Mont-Ventoux regional nature park, France. Reg Environ Change.

[CR117] United Nations Development Programme (UNDP) (2020a) Technical notes: calculating the human development indices-graphical presentation. United Nations Development Programme (UNDP)

[CR118] United Nations Development Programme (UNDP) (2020b) Human Development Report 2020: the next frontier: human development and the Anthropocene. United Nations Development Programme (UNDP), New York

[CR119] Vuong Q-H (2018). The (ir)rational consideration of the cost of science in transition economies. Nat Hum Behav.

[CR120] Vuong Q-H (2020). Reform retractions to make them more transparent. Nature.

[CR121] Williams SE, Hobday AJ, Falconi L, Hero JM, Holbrook NJ, Capon S, Bond NR, Ling SD, Hughes L (2020). Research priorities for natural ecosystems in a changing global climate. Glob Change Biol.

