# Supplementary material for: Perspectives on socio-ecological studies in the Northern and Southern Hemispheres
Source: Humanit Soc Sci Commun. 2023 Feb 20;10(1):66. doi: 10.1057/s41599-023-01545-w (PMC9940092; doi:10.1057/s41599-023-01545-w)
Supplement: Supplementary file 1 — Supplementary information [file 41599_2023_1545_MOESM1_ESM.docx]

**Supplementary Information**

**Annex 1**. Number (*n*) of reviewed socio-ecological studies per country based on the Human Development Index (HDI).

| **Hemisphere** | **Country** | **HDI value** | **Code** | ***n* Scopus paper results** | ***n* paper selected** |
| --- | --- | --- | --- | --- | --- |
| South | Australia | 0.944 | AUS | 87 | 47 |
|  | New Zealand | 0.931 | NZL | 22 | 16 |
|  | Chile | 0.851 | CHL | 45 | 35 |
|  | Argentina | 0.845 | ARG | 30 | 21 |
|  | Uruguay | 0.817 | URY | 4 | 2 |
|  | Botswana | 0.735 | BWA | 9 | 7 |
|  | South Africa | 0.709 | ZAF | 49 | 32 |
| North | Norway | 0.957 | NOR | 8 | 8 |
|  | Ireland | 0.955 | IRL | 10 | 3 |
|  | Switzerland | 0.950 | SWI | 5 | 3 |
|  | Iceland | 0.949 | ISL | 4 | 1 |
|  | Hong Kong | 0.949 | HKG | 4 | 2 |
|  | Germany | 0.947 | DEU | 20 | 11 |
|  | Sweden | 0.945 | SWE | 14 | 7 |
|  | Netherlands | 0.944 | NLD | 12 | 6 |
|  | Denmark | 0.940 | DNK | 6 | 2 |
|  | Finland | 0.938 | FIN | 8 | 3 |
|  | United Kingdom | 0.932 | GBR | 19 | 12 |
|  | Belgium | 0.931 | BEL | 5 | 3 |
|  | Canada | 0.929 | CAN | 46 | 24 |
|  | United States | 0.926 | USA | 111 | 69 |
|  | Austria | 0.922 | AUT | 8 | 3 |
|  | Japan | 0.919 | JPN | 41 | 14 |
|  | Luxembourg | 0.916 | LUX | 1 | 1 |
|  | South Korea | 0.916 | KOR | 3 | 2 |
|  | Spain | 0.904 | ESP | 61 | 44 |
|  | France | 0.901 | FRA | 26 | 20 |
|  | Kazakhstan | 0.825 | KAZ | 1 | 1 |
|  | Russia | 0.824 | RUS | 13 | 11 |
|  | Turkey | 0.820 | TUR | 3 | 2 |
|  | Georgia | 0.812 | GEO | 3 | 2 |
|  | China | 0.761 | CHN | 91 | 53 |
| Total |  |  |  | 769 | 467 |

HDI values for 2019 in the Human Development Report 2020. The review of studies in Scopus ended on October 28, 2021.

**Annex 2.** Papers reviewed by country.

| **Country searched** | **Year** | **Tittle** | **Authors** |
| --- | --- | --- | --- |
| Argentina | 2010 | Applying the Ecosystem Approach to Select Priority Areas for Forest Landscape Restoration in the Yungas, Northwestern Argentina | Ianni, Geneletti, D. |
|  | 2013 | Managing Socio-Ecological Systems to Achieve Sustainability: A Study of Resilience and Robustness | Domptail, S., et al. |
|  | 2015 | Is it advisable to further promote forest plantations in northern Patagonia Argentina? Where, why and for whom? | Paruelo, J. |
|  | 2015 | Linking marine and terrestrial ecosystem services through governance social networks analysis in Central Patagonia (Argentina) | Alonso Roldán, V., et al. |
|  | 2016 | Focusing conservation efforts on ecosystem service supply may increase vulnerability of socio-ecological systems | Laterra, P., et al. |
|  | 2016 | Participatory scenario planning for developing innovation in community adaptation responses: three contrasting examples from Latin America | Brown, I., et al. |
|  | 2018 | Is It Possible to Completely Adapt Agriculture Production to the Effects of Climate Variability and Change in Central Argentina? New Approaches in Face of New Challenges | Wehbe, M.B., et al. |
|  | 2018 | Human-carnivore interaction in a context of socio-productive crisis: Assessing smallholder strategies for reducing predation in North-west Patagonia, Argentina | Gáspero, P.G., et al. |
|  | 2018 | Potential associations between the mite Varroa destructor and other stressors in honeybee colonies (*Apis mellifera* L.) in temperate and subtropical climate from Argentina | Giacobino, A., et al. |
|  | 2018 | A co-designed, transdisciplinary adaptive management framework for artisanal fisheries of Pehuen Co and Monte Hermoso (Argentina) | Berninsone, L.G., et al. |
|  | 2018 | From traditional knowledge to novel adaptations of transhumant pastoralists the in face of new challenges in North Patagonia | Easdale, M.H., Aguiar, M.R. |
|  | 2019 | Is collaborative management always possible? The case of Sauce Grande river basin, Argentina | Zilio, M.I., et al. |
|  | 2020 | Analysis of the socio-ecological system of the Bahía Blanca estuary (Argentina) and its impact on ecosystem services and human well-being | Speake, M.A., et al. |
|  | 2021 | The role of scientists at the human-nature interface on mab protected areas | López, L., et al. |
|  | 2021 | Assessing Socio-ecological Systems Using Social Media Data: An Approach for Forested Landscapes in Tierra del Fuego, Argentina | Huertas Herrera, A., et al. |
|  | 2021 | Perspectives regarding the contributions and states of native forest by stakeholders linked to livestock production in the entre rios espinal | Rojido, I.J., et al. |
|  | 2021 | Can participatory action research foster social learning in communities struggling for land tenure? | Below, J.V., et al. |
|  | 2021 | Controversies and Common Ground in Wild and Domestic Fine Fiber Production in Argentina | von Thungen, J., et al. |
|  | 2021 | Traditional puesteros’ perceptions of biodiversity in semi-arid Southern Mendoza, Argentina | Llano, C., et al. |
|  | 2021 | Agroecology in semiarid Chaco forests of Argentina: transdisciplinary analysis of a sustainable peasant farm | Cotroneo S M, et al. |
|  | 2021 | Human-wildlife conflicts in the southern yungas: What role do raptors play for local settlers? | Salom, A., et al. |
| Australia | 2004 | Ross river virus disease in Australia: Epidemiology, socioecology and public health response | Tong, S. |
|  | 2007 | Conceptualizing and operationalizing social resilience within commercial fisheries in northern Australia | Marshall, N.A., Marshall, P.A. |
|  | 2007 | Linking land to ocean: Feedbacks in the management of socio-ecological systems in the Great barrier Reef catchments | Gordon, I.J. |
|  | 2008 | Community-based research: Facilitating sustainability learning | Thomsen, D.C. |
|  | 2008 | Applying the sustainable livelihoods approach in Australian desert Aboriginal development | Davies, J., et al. |
|  | 2008 | Interdependence of common-pool resources: Lessons from a set of nested catchments in Australia | Sarker, A., et al. |
|  | 2009 | Building and managing resilience in community-based NRM groups: An Australian case study | Gooch, M., Warburton, J |
|  | 2011 | Regional planning and resilient futures: Destination modelling and tourism development-the case of the ningaloo coastal region in Western Australia | Tod, J., et al. |
|  | 2011 | Stakeholder engagement in social learning to resolve controversies over land-use change to plantation forestry | Leys, A.J., Vanclay, J.K. |
|  | 2011 | An integrated decision-support approach in prioritizing risks of non-indigenous species in the face of high uncertainty | Liu, S., et al. |
|  | 2011 | Appreciating Institutional Complexity in Water Governance Dynamics: A Case from the Murray-Darling basin, Australia | Wallis, P.J., Ison, R.L. |
|  | 2012 | Transformational capacity and the influence of place and identity | Marshall, N.A., et al. |
|  | 2012 | Conceptualizing Work, Family and Community: A Socio-Ecological Systems Model, Taking Account of Power, Time, Space and Life Stage | Pocock, B., et al. |
|  | 2013 | Decolonising property: Exploring ethics, land, and time, through housing interventions in contemporary Australia | Crabtree, L. |
|  | 2013 | Using a Cognitive Mapping Approach to Frame the Perceptions of Water Users About Managing Water Resources: A Case Study in the Australian Capital Territory | ElSawah, S., et al. |
|  | 2013 | Social Vulnerability of marine Resource Users to Extreme Weather Events | Marshall, N.A., et al. |
|  | 2013 | Climate change and indigenous natural resource management: A review of socio-ecological interactions in the Alinytjara Wilurara NRM region | Wiseman, N.D., Bardsley, D.K. |
|  | 2014 | Modelling the benefits of habitat restoration in socio-ecological systems | Jellinek, S., et al. |
|  | 2015 | Assessment of vulnerability to climate change using a multi-criteria outranking approach with application to heat stress in Sydney | El-Zein, A., Tonmoy, F.N. |
|  | 2015 | Indigenous biocultural knowledge in ecosystem science and management: Review and insight from Australia | Ens, E.J., et al. |
|  | 2015 | Preventing weed spread: A survey of lifestyle and commercial landholders about *Nassella trichotoma* in the Northern Tablelands of New South Wales, Australia | Ruttledge, A., et al. |
|  | 2015 | Managing tree plantations as novel socioecological systems: Australian and North American perspectives | Lindenmayer, D., et al. |
|  | 2015 | The pace and progress of adaptation: marine climate change preparedness in Australia's coastal communities | Bradley, M., et al. |
|  | 2015 | Organizational drivers that strengthen adaptive capacity in the coastal zone of Australia | Dutra, L.X.C., et al. |
|  | 2015 | Social and institutional factors influencing industry driven area-wide fruit fly management in Australian horticultural industries | Kruger, H.P. |
|  | 2016 | Monitoring to Learn, Learning to Monitor: A Critical Analysis of Opportunities for Indigenous Community-Based Monitoring of Environmental Change in Australian Rangelands | Wiseman, N.D., Bardsley, D.K. |
|  | 2016 | Returns from matching management resolution to ecological variation in a coral Reef fishery | Bode, M., et al. |
|  | 2016 | Objectives for management of socio-ecological systems in the Great barrier Reef region, Australia | Van Putten, I.E., et al. |
|  | 2016 | Principles for operationalizing climate change adaptation strategies to support the resilience of estuarine and coastal ecosystems: An Australian perspective | Sheaves, M., et al. |
|  | 2016 | The effect of individual and social environments on the users thermal perceptions of educational urban precincts | Shooshtarian, S., Ridley, I. |
|  | 2016 | Exploring Local Responses to a Wicked Problem: Context, Collective Action, and Outcomes in Catchments in Subtropical Australia | Patterson, J.J. |
|  | 2017 | The effect of physical and psychological environments on the users thermal perceptions of educational urban precincts | Shooshtarian, S., Ridley, I. |
|  | 2017 | Study of thermal satisfaction in an Australian education precinct | Shooshtarian, S., Rajagopalan, P. |
|  | 2018 | An integrated risk-assessment framework for multiple threats to floodplain values in the Kakadu Region, Australia, under a changing climate | Bayliss, P., et al. |
|  | 2018 | The role of Great barrier Reef tourism operators in addressing climate change through strategic communication and direct action | Goldberg, J., et al. |
|  | 2018 | Vulnerability to sea level rise: A novel local-scale indicator-based assessment methodology and application to eight beaches in Shoalhaven, Australia | Tonmoy, F.N., El-Zein, A |
|  | 2018 | Poorly-designed goals and objectives in resource management plans: Assessing their impact for an Ecosystem-Based Approach to marine Spatial Planning | Domínguez-Tejo, E., Metternicht, G. |
|  | 2018 | Changing Water system vulnerability in Western Australia's Wheatbelt region | Boruff, B., et al. |
|  | 2018 | Towards a socio-ecological framework to address gender inequity in computer science | Michell, D., et al. |
|  | 2018 | Modelling the Great Australian Bight Ecosystem | Fulton, E.A., et al. |
|  | 2018 | Strategies for developing transformative capacity in urban Water management sectors: The case of Melbourne, Australia | Brodnik, C., Brown, R. |
|  | 2019 | Are we there yet? The Murray-Darling basin and sustainable Water management | Pittock, J. |
|  | 2019 | Losing the authority–what institutional architecture for cooperative governance in the Murray Darling basin? | Alexandra, J. |
|  | 2019 | Socio-ecological mechanisms for persistence of native Australian grasses under pressure from nitrogen runoff and invasive species | Thampi, V.A., et al. |
|  | 2020 | Research priorities for natural ecosystems in a changing global climate | Williams, S.E., et al. |
|  | 2020 | Widening the lens: Understanding urban parks as a network | Torabi, N., et al. |
|  | 2021 | Now it's not a billabong' Eco-cultural assessment of billabong condition in remote northern Australia | Russell, S., et al. |
| Austria | 2009 | Combining agent-based and stock-flow modelling approaches in a participative analysis of the integrated land system in Reichraming, Austria | Gaube, V., et al. |
|  | 2013 | Impact of urban planning on household's residential decisions: An agent-based simulation model for Vienna | Gaube, V., Remesch, A. |
|  | 2021 | Simulating the forest fuel market as a socio-ecological system with spatial agent-based methods: A case study in Carinthia, Austria | Scholz, J., et al. |
| Belgium | 2013 | Garden management and soil fertility in Flemish domestic gardens | Dewaelheyns, V., et al. |
|  | 2014 | Towards participatory integrated valuation and modelling of ecosystem services under land-use change | Fontaine, C.M., et al. |
|  | 2021 | Capabilities, Ecosystem Services, and Strong Sustainability through SMCE: The Case of Haren (Belgium) | Pelenc, J., Etxano, I. |
| Botswana | 2002 | Learning, life history, and productivity_Children’s lives in the Okavango Delta, Botswana | Bock, J. |
|  | 2014 | Prey mortality profiles indicate that Early Pleistocene Homo at Olduvai was an ambush predator | Bunn, H.T., Gurtov, A.N. |
|  | 2016 | Fluid waters and rigid livelihoods in the Okavango Delta of Botswana | King, B., et al. |
|  | 2018 | Livelihood Dynamics Across a Variable Flooding Regime | King, B., et al. |
|  | 2019 | Political Ecologies of Dynamic Wetlands: Hydrosocial Waterscapes in the Okavango Delta | King, B., et al. |
|  | 2019 | Complex interactions between climate change, sanitation, and groundwater quality: a case study from Ramotswa, Botswana | McGill, B.M., et al. |
|  | 2019 | Adaptation strategies to environmental and policy change in semi-arid pastoral landscapes: Evidence from Ngamiland, Botswana | Basupi, L.V., et al. |
| Canada | 2004 | Multiple intervention research programs in community health | Edwards, N., et al.. |
|  | 2010 | A social-spatial approach to ecological governance | Tyler, M.-E., Quinn, M. |
|  | 2013 | The potential use of agroforestry community gardens as a sustainable import-substitution strategy for enhancing food security in subarctic Ontario, Canada | Spiegelaar, N.F., Tsuji, L.J.S., Oelbermann, M. |
|  | 2013 | The Dynamic Multiscale Nature of Climate Change Vulnerability: An Inuit Harvesting Example | Ford, J.D., et al. |
|  | 2014 | Environmental Function Analysis: A decision support tool for integrated sandy beach planning | Amyot, J., Grant, J. |
|  | 2015 | Data and information management for the monitoring of biodiversity in Alberta | Sõlymos, P., et al. |
|  | 2016 | Inuit knowledge and environmental assessment in Nunavut, Canada | Gondor, D. |
|  | 2016 | Monitoring Nûtimesânân Following the Diversion of Our River: A Community-led Registry in Eeyou Istchee, Northern Québec | Strangway, R.E., et al. |
|  | 2016 | Hegemonic and emerging concepts of conservation: a critical examination of barriers to incorporating Indigenous perspectives in protected area conservation policies and practice | Shultis, J., Heffner, S. |
|  | 2016 | Interaction of ecological and angler processes: Experimental stocking in an open access, spatially structured fishery | Mee, J.A., et al. |
|  | 2017 | Integrative analysis of the Lake Simcoe watershed (Ontario, Canada) as a socio-ecological system | Neumann, A., et al. |
|  | 2017 | Representation of local urban forestry issues in Canadian newspapers: Impacts of a major ice storm | Conway, T.M., Jalali, M.A. |
|  | 2017 | Dog-bites, rabies and One Health: Towards improved coordination in research, policy and practice | Rock, M.J., et al. |
|  | 2018 | Multistate matrix population model to assess the contributions and impacts on population abundance of domestic cats in urban areas including owned cats, unowned cats, and cats in shelters | Flockhart, D.T.T., Coe, J.B. |
|  | 2018 | Decolonizing Urban Political Ecologies: The Production of Nature in Settler Colonial Cities | Simpson, M., Bagelman, J. |
|  | 2018 | The use of context-based environmental indicators in corporate reporting | Haffar, M., Searcy, C. |
|  | 2018 | Coupling stated preferences with a hydrological water resource model to inform water policies for residential areas in the Okanagan Basin, Canada | Conrad, S.A., Yates, D. |
|  | 2019 | Scaling up research-for-development innovations in food and agricultural systems | Shilomboleni, H., De Plaen, R. |
|  | 2020 | Transforming conflict over natural resources: A socio-ecological systems analysis of agricultural drainage | Minnes, S., et al. |
|  | 2020 | ‘I think it is the toughest animal in the North’: human-wolverine interactions among hunters and trappers in the Canadian Northwest Territories | Bonamy, M., et al. |
|  | 2020 | Characterizing Exposure to and Sharing Knowledge of Drivers of Environmental Change in the St. Lawrence System in Canada | Beauchesne, D., et al. |
|  | 2020 | Investigating local concerns regarding large mammal restoration: group size in a growing population of reintroduced bison (Bison) | Jung, T.S. |
|  | 2021 | Action research to improve water quality in Canada’s Rideau Canal: how do local groups reshape environmental governance? | Mistry, I., et al. |
|  | 2021 | A systems perspective analysis of an increased use of forest bioenergy in Canada: Potential carbon impacts and policy recommendations | Giuntoli, J., et al. |
| Chile | 2011 | A methodology for community engagement in the introduction of renewable based smart microgrid | Alvial-Palavicino, C., et al. |
|  | 2012 | Synanthropy and marine conservation: The case of the marine otter lontra felina in Southern Chile | Cursach, J.A., et al. |
|  | 2012 | Influence of landscape change on nearshore fisheries in southern Chile | Holt, T.V., et al. |
|  | 2015 | Knowledge Dialogue to Attain Global Scientific Excellence and Broader Social Relevance | Anderson, C.B., et al. |
|  | 2015 | Applying an ecosystem service approach to unravel links between ecosystems and society in the coast of central Chile | de Juan, S., et al. |
|  | 2015 | Affordance of landscapes and economic socio-spatial networks in the quinchao archipelago, chile: A contribution to landscape research and island studies | Hidalgo, C., et al. |
|  | 2015 | The firewood dilemma: Human health in a broader context of well-being in Chile | Reyes, R., et al. |
|  | 2016 | Sustainable development? Salmon aquaculture and late modernity in the archipelago of chiloé, Chile | Barton, J.R., Román, Á. |
|  | 2016 | Personal reflections on natural history as common ground for interdisciplinary multispecies socio-ecological research | Root-Bernstein, M. |
|  | 2017 | Biocultural homogenization in Urban settings: Public knowledge of birds in city parks of Santiago, Chile | Celis-Diez, J.L., et al. |
|  | 2017 | Face-to-Face with the caracara”: A proposal to reconnect people and nature using birding | Cristóbal Pizarro, J., et al. |
|  | 2017 | Physiological and histopathological impacts of increased carbon dioxide and temperature on the scallops *Argopecten purpuratus* cultured under upwelling influences in northern Chile | Lardies, M.A., et al. |
|  | 2018 | Assessment of the ecosystem service of Water regulation under scenarios of conservation of native vegetation and expansion of forest plantations in south-central Chile | Jullian, C., et al. |
|  | 2018 | The interplay between fish farming and nature based recreation-tourism in Southern Chile: A perception approach | Outeiro, L., et al. |
|  | 2018 | Two thousand years of land-use and vegetation evolution in the andean highlands of northern chile inferred from pollen and charcoal analyses | Domic, A.I., et al. |
|  | 2018 | Water governance and emerging challenges for rigid normative and institutional structures: An analysis from the chilean case | Galvis, L.K.S., et al. |
|  | 2019 | Livelihood trajectories in the Chilean Patagonian region: an ethnographic approach to coastal and marine socioecological change | Mellado, M.A., et al. |
|  | 2019 | Geohistorical records of the Anthropocene in Chile | Gayo E.M., et al. |
|  | 2019 | Paisajes en transición: Gradientes urbano-rurales y antropización del bosque templado andino del sur de Chile | Vergara, G., Ibarra, J.T. |
|  | 2019 | Exploring determinants for the implementation of mixed TURF-aquaculture systems | Sepúlveda, C., et al. |
|  | 2019 | Social networks analysis in a socio-ecological estuarine system of southern Chile (41,6°S): Diagnosis and contributions for the improvement of governance in riparian contexts | Andrade, L., et al. |
|  | 2020 | Moving beyond co-management: Opportunities and limitations for enabling transitions to polycentric governance in chile’s territorial user rights in fisheries policy | Ebel, S.A. |
|  | 2020 | Territorial complexity in fishermen's coves of Chiloé (Chile): contributions for coastal management | Ther-Rios, F., et al. |
|  | 2020 | Local knowledge in montane homegardens in the southern Andes: A refuge of Mapuche Pewenche biocultural memory | Santiago, C.M., et al. |
|  | 2020 | Community responses in desert areas after extreme weather events in Northern Chile | Cepeda, J.F.L., et al. |
|  | 2020 | Social Underpinnings of Ecological Knowledge: Business Perceptions of Biodiversity as Social Learning | Smith, T., et al. |
|  | 2020 | Exploring perceived well-being from urban parks: Insights from a megacity in latin America | Parra-Saldívar, A., et al. |
|  | 2020 | Conservation of Rapa Nui Waters strongly supported by publications in aquatic Conservation | Gaymer, C.F., Aburto, J.A. |
|  | 2020 | Territory and terroir cases of small-scale wine production in the central south part of Chile | Aguayo, B.E.C., et al. |
|  | 2020 | Land use as a socio-ecological system: Developing a transdisciplinary approach to studies of land use change in South-Central Chile (Book Chapter) | Manuschevich, D. |
|  | 2021 | Who has benefited? A socio-ecological chronology of urban resilience in the early reconstruction of talca after the 27-F earthquake, Chile 2010-2012 | Contardo, J.I., Figueroa, P.M. |
|  | 2021 | Perception of environmental quality in a beach of high social segregation in northern Chile: Importance of social studies for beach conservation | González, S.A., et al. |
|  | 2021 | Unravelling non-human agency in sustainability transitions | Contesse, M., et al. |
|  | 2021 | At the crossroads of co-management in a complex socioecological system: Lessons from the first participatory management plan in Chile | Barahona, N., Molinet, C. |
|  | 2021 | Residential sidewalk gardens and biological conservation in the cities: Motivations and preferences that guide the floristic composition of a little-explored space | Guerrero-Leiva, N., et al. |
| China | 2004 | Socioecological transformation triggered by national rubber plantations in Yunnan, China: The impact of Han-chinese immigration into Xishuangbanna since the 1950s | Fukao, Y. |
|  | 2009 | Hydropower and sustainability: Resilience and vulnerability in China's powersheds | McNally, A., et al. |
|  | 2010 | Rangeland degradation on the Qinghai-Tibetan plateau: A review of the evidence of its magnitude and causes | Harris, R.B. |
|  | 2012 | An assessment of China's ecological environment quality change and its spatial variation | Sun, D., et al. |
|  | 2013 | Spatial analysis of China's eco-environmental quality: 1990-2010 | Sun, D., et al. |
|  | 2013 | Analyzing land-use change in farming-pastoral transitional region using autologistic model and household survey approach | Zhu, L., et al. |
|  | 2014 | Applying the concept of spatial resilience to socio-ecological systems in the urban wetland interface | Li, Y., et al. |
|  | 2014 | Effects of land use change on soil carbon storage and water consumption in an oasis-desert ecotone | Lü, Y., et al. |
|  | 2014 | Governing forest restoration: Local case studies of sloping land conversion program in Southwest China | He, J. |
|  | 2015 | Using Pléiades data to understand and monitor a dynamic socio-ecological system: China's Poyang Lake | Huber, C., et al. |
|  | 2015 | Study on measurement and impact mechanism of socio-ecological system resilience in Qiandao Lake | Wang, Q., et al. |
|  | 2015 | A basic assessment of residential plant diversity and its ecosystem services and disservices in Beijing, China | Wang, H.-F., et al. |
|  | 2016 | Integrating the spatial proximity effect into the assessment of changes in ecosystem services for biodiversity conservation | Liu, Y., et al. |
|  | 2017 | Quantifying urban ecological governance: A suite of indices characterizes the ecological planning implications of rapid coastal urbanization | Li, Y., et al. |
|  | 2017 | Quantitative evaluation of desertification restoration based on the social-ecological system: A case study in Yanchi, Ningxia Hui Autonomous Region | Hou, C.X., et al. |
|  | 2017 | Biocapacity optimization in regional planning | Guo, J., et al. |
|  | 2017 | Spatial-temporal changes of coastal and marine disasters risks and impacts in Mainland China | Fang, J., et al. |
|  | 2017 | Assessing the impacts of human activities and climate variations on grassland productivity by partial least squares structural equation modeling (PLS-SEM) | Sha, Z., et al. |
|  | 2017 | Hydrogeomorphic Ecosystem Responses to Natural and Anthropogenic Changes in the Loess Plateau of China | Fu, B., et al. |
|  | 2018 | An evaluation index system of vulnerability of the desertification reversion process based on the socio-ecological systems theory | Wang, Y., et al. |
|  | 2018 | Fengshui forests and village landscapes in China: Geographic extent, socioecological significance, and conservation prospects | Chen, B., et al. |
|  | 2018 | Conservation and development in conflict: Regeneration of wild Davidia involucrata (Nyssaceae) communities weakened by bamboo management in south-central China | Qian, S., et al. |
|  | 2018 | Optimizing policy for balanced industrial profit and water pollution control under a complex socioecological system using a multiagent-based model | Deng, C., et al. |
|  | 2018 | Evaluation on dynamic change and interrelations of ecosystem services in a typical mountain-oasis-desert region | Wei, H., et al. |
|  | 2018 | Taking an ecosystem services approach for a new national park system in China | He, S., et al. |
|  | 2019 | Ecological study on the value-creation mechanism of farmer profession cooperatives | Zhang, J., He, Y. |
|  | 2019 | How societal values determine the local use of forest resources-findings from the rural community Kegong (Northwest Yunnan, China) | Nassl, M., Löffer, J. |
|  | 2019 | Vulnerability assessment of urban socio-ecological systems in coastal zones under the influence of typhoons:Big data perspective | Wu, W., et al. |
|  | 2019 | A spatio-temporal delineation of trans-boundary ecosystem service flows from Inner Mongolia | Xie, G., et al. |
|  | 2019 | Spatial correlations among ecosystem services and their socio-ecological driving factors: A case study in the city belt along the Yellow River in Ningxia, China | Lyu, R., et al. |
|  | 2019 | Socio-ecological changes on the Loess Plateau of China after Grain to Green Program | Wu, X., et al. |
|  | 2019 | Spatio-temporal evolution and impact mechanism of socioecological system vulnerability in poor mountainous tourist distinations: Taking Dabie Mountain Area as example | Wang, Q., et al. |
|  | 2019 | Evaluating poverty alleviation by relocation under the link policy: A case study from Tongyu County, Jilin Province, China | Zou, C., et al. |
|  | 2019 | Regime shift and redevelopment of a mining area’s socio-ecological system under resilience thinking: a case study in Shanxi Province, China | Yang, Y., et al. |
|  | 2019 | Does the "returning farmland to forest program" drive community-level changes in landscape patterns in China? | Li, W., et al. |
|  | 2020 | Integrated assessment of land-use/coverage changes and their impacts on ecosystem services in Gansu Province, northwest China: implications for sustainable development goals | Liu, L., et al. |
|  | 2020 | Evaluating fisheries conservation strategies in the socio-ecological system: A grid-based dynamic model to link spatial conservation prioritization tools with tactical fisheries management | Li, Y., et al. |
|  | 2020 | The effects of farmland use rights trading and labor outmigration on the governance of the irrigation commons: Evidence from China | Su, Y., et al. |
|  | 2020 | Diversity and Adaptation in Local Forest Governance in Yunnan, China | Brown, M. |
|  | 2020 | Socio-ecological determinants on spatio-temporal changes of groundwater in the Yellow River Basin, China | Lin, M., et al. |
|  | 2020 | Ecological response to urban development in a changing socio-economic and climate context: Policy implications for balancing regional development and habitat conservation | Huang, J., et al. |
|  | 2020 | Spatio-temporal quantification of patterns, trade-offs and synergies among multiple hydrological ecosystem services in different topographic basins | Sun, X., et al. |
|  | 2020 | Farmers’ livelihood adaptability in rural tourism destinations: An evaluation study of rural revitalization in China | Li, H., et al. |
|  | 2020 | Village fengshui forests as forms of cultural and ecological heritage: Interpretations and conservation policy implications from southern China | Chen, J., et al. |
|  | 2021 | Identifying multiple stakeholders’ roles and network in urban waste  separation management-a case study in Xiamen, China | Lishan, X., et al. |
|  | 2021 | Synergizing a socio-ecological system: reflections on community-based natural resource management at the World Heritage Site of Mount Huangshan, China | Li, J., et al. |
|  | 2021 | Dynamics of spatial relationships among ecosystem services and their determinants: Implications for land use system reform in Northwestern China | Lyu, R., et al. |
|  | 2021 | Nonlinearity and threshold effects of landscape pattern on water quality in a rapidly urbanized headwater watershed in China | Liu, J., et al. |
|  | 2021 | Spatial identification and determinants of trade-offs among multiple land use functions in Jiangsu Province, China | Fan, Y., et al. |
|  | 2021 | Perceptions of Local People toward Wild Edible Plant Gathering and Consumption: Insights from the Q-method in Hani Terraces | Ding, L.,et al. |
|  | 2021 | Reframing water-related ecosystem services flows | Lin, J., et al. |
|  | 2021 | Role of social networks in building household livelihood resilience under payments for ecosystem services programs in a poor rural community in China | Wang, Y., et al. |
|  | 2021 | Do Forest Landscape Pattern Planning and Optimization Play a Role in Enhancing Soil Conservation Services in Mountain Areas of Western China? | Gong, J.,et al. |
| Denmark | 2014 | The socio-ecological dimensions of hydrocarbon development in the Disko Bay region of Greenland: Opportunities, risks, and tradeoffs | McDowell, G., Ford, J.D. |
|  | 2017 | Transitions of social-ecological subsistence systems in the Arctic | Fauchald, P., et al. |
| Finland | 2008 | Addressing multiple goals of ecosystem approach through deliberation and technology development | Varjopuro, R. |
|  | 2014 | What is the socio-political scaffolding CCS needs to thrive? case study from finland | Toikka, A., et al. |
|  | 2019 | Safe places: Increasing Finnish waterfowl resilience through human-made wetlands | Mustonen, T., Kontkanen, H. |
| France | 2008 | Including multiple differing stakeholder values into vulnerability assessments of socio-ecological systems | de Chazal, J., et al. |
|  | 2012 | Modelling carcass disposal practices: Implications for the management of an ecological service provided by vultures | Dupont, H., et al. |
|  | 2013 | Socio-ecological adaptation to climate change: A comparative case study from the Mediterranean wine industry in France and Australia | Lereboullet, A.-L., et al. |
|  | 2013 | Taking into account farmers' decision making to map fine-scale land management adaptation to climate and socio-economic scenarios | Lamarque, P., et al. |
|  | 2015 | The Crau Plain (West of Provence, France), a territory with economic and ecological issues in mutation | Beltrando, G. |
|  | 2016 | Protected areas and their surrounding territory: Socioecological systems in the context of ecological solidarity | Mathevet, R., et al. |
|  | 2016 | Socio-ecological transitions toward low-carbon port cities: trends, changes and adaptation processes in Asia and Europe | Mat, N., et al. |
|  | 2016 | The contribution of agent-based simulations to conservation management on a Natura 2000 site | Dupont, H., et al.. |
|  | 2017 | Managing marine socio-ecological systems: Picturing the future | Thébaud, O., et al. |
|  | 2017 | The Crau plain (French mediterranean coast): An uncertain future in the context of climate chang | Beltrando, G. |
|  | 2018 | Towards sustainable and multifunctional agriculture in farmland landscapes: Lessons from the integrative approach of a French LTSER platform | Bretagnolle, V., et al. |
|  | 2018 | Dealing with impact. An interdisciplinary, multi-site ethnography of environmental impact assessment in the coastal zone | Mazé, C., et al. |
|  | 2018 | Policy instruments and political dynamics of adaptations to global change in coastal areas | Dhénain, S., Barreteau, O. |
|  | 2018 | RECOTOX, a French initiative in ecotoxicology-toxicology to monitor, understand and mitigate the ecotoxicological impacts of pollutants in socioagroecosystems | Mougin, C., et al. |
|  | 2019 | Towards an Integrated Framework for the Governance of a Territorialised Agroecological Transition | Triboulet, P., et al. |
|  | 2019 | The effect of stumpage prices on large-area forest growth forecasts based on socio-ecological models | Fortin, M., et al. |
|  | 2021 | Historical ecology and ancient forests: Progress, conservation issues and scientific prospects, with some examples from the French case | Bergès, L., Dupouey, J.-L. |
|  | 2021 | Adaptive measures for mountain Mediterranean forest ecosystem services under climate and land cover change in the Mont-Ventoux regional nature park, France | Tuffery, L., et al. |
|  | 2021 | Game of Cruxes: co‑designing a game for scientists and stakeholders for identifying joint problems | Nicolas, S., et al. |
|  | 2021 | The extended concept of littoral active zone considering soft sediment shores as social-ecological systems, and an application to Brittany (North-Western France) | Fanini, L., et al. |
| Georgia | 2016 | Ethno-ecological contexts of the Skhalta Gorge and the Upper Svaneti (Georgia, the Caucasus) | Tevzadze, G., Kikvidze, Z. |
|  | 2019 | Land-use change related to topography and societal drivers in high-mountains - A case study in the upper watershed of the Tergi (Kazbegi Region), Greater Caucasus | Theissen, T., et al. |
| Germany | 1998 | Socioecology of the drug problem | Tretter, F. |
|  | 2012 | A socio-ecological adaptive approach to contaminated mega-site management: From 'control and correct' to 'coping with change' | Schirmer, M., et al. |
|  | 2015 | Supporting SLEUTH - Enhancing a cellular automaton with support vector machines for urban growth modeling | Rienow, A., Goetzke, R. |
|  | 2015 | Cross-sectoral resource management: How forest management alternatives affect the provision of biomass and other ecosystem services | Villamayor-Tomas, S., et al. |
|  | 2015 | Sharing the world with mammoths, cave lions and other beings: Linking animal-human interactions and the Aurignacian "belief world" | Hussain, S.T., Floss, H. |
|  | 2016 | Urban gray vs. urban green vs. soil protection — Development of a systemic solution to soil sealing management on the example of Germany | Artmann, M. |
|  | 2017 | Using the concepts of green infrastructure and ecosystem services to specify leitbilder for compact and green cities-The example of the landscape plan of Dresden (Germany) | Artmann, M., et al. |
|  | 2018 | Spatial correlation of agri-environmental measures with high levels of ecosystem services | Frueh-Mueller, A., et al.. |
|  | 2018 | Model-based evaluation of urban river restoration: Conflicts between sensitive fish species and recreational users | Zingraff-Hamed, A., et al. |
|  | 2020 | Urban green spaces for the social interaction, health and well-being of older people— An integrated view of urban ecosystem services and socio-environmental justice | Enssle, F., Kabisch, N. |
|  | 2021 | A methodological framework for the assessment of regulating and recreational ecosystem services in urban parks under heat and drought conditions | Kabisch, N., et al. |
| Hong Kong | 2016 | A multi-objective optimization approach for health-care facility location-allocation problems in highly developed cities such as Hong Kong | Zhang, W., et al. |
|  | 2019 | Resilience of an inshore fishing population in Hong Kong: Paradox and potential for sustainable fishery policy | Patchell, J., Cheng, C. |
| Iceland | 2014 | Late-Holocene land surface change in a coupled social-ecological system, southern Iceland: A cross-scale tephrochronology approach | Streeter, R., Dugmore, A. |
| Ireland | 2010 | The potential role of Sustainability Science in coastal zone management | Cummins, V., McKenna, J. |
|  | 2020 | Ecosystems of educational disadvantage: Supporting children and young people receiving child protection and welfare services in Ireland | Flynn, S. |
|  | 2020 | Contextualising missed care in two healthcare inquiries using a socio-ecological systems approach | Phelan, A., Kirwan, M. |
| Japan | 2014 | Using sustainability science to analyse social–ecological restoration in NE Japan after the great earthquake and tsunami of 2011 | Takeuchi, K., et al. |
|  | 2016 | To tweet or not to tweet: Factors affecting the intensity of twitter usage in Japan and the online and offline sociocultural norms | Wang, S.S. |
|  | 2017 | The Potential Role of Tree Diversity in Reducing Shallow Landslide Risk | Kobayashi, Y., Mori, A.S. |
|  | 2018 | A problem of social fit? Assessing the role of bridging organizations in the recoupling of socio-ecological systems | Boakye-Danquah, J., et al. |
|  | 2018 | ‘The tragedy of the commons’ by underuse: Toward a conceptual framework based on ecosystem services and satoyama perspective | Miyanaga, K., Shimada, D. |
|  | 2019 | Investigating future ecosystem services through participatory scenario building and spatial ecological–economic modellin | Kabaya, K., et al. |
|  | 2019 | Development of land-use scenarios using vegetation inventories in Japan | Shoyama, K., et al. |
|  | 2019 | Oysters and Tsunami: Iterative Learning and Nested Governance as Resilience in Post-Disaster aquaculture in Hokkaido, Japan | Ito, T., Watanabe, T. |
|  | 2020 | Changes in the potential stocks of coral Reef ecosystem services following coral bleaching in Sekisei Lagoon, southern Japan: implications for the future under global warming | Sato, M., et al. |
|  | 2020 | Building resilient socio-ecological systems in Japan: Satoyama examples from Shiga Prefecture | Fukamachi, K. |
|  | 2021 | Projecting population distribution under depopulation conditions in Japan: scenario analysis for future socio-ecological systems | Hori, K., et al. |
|  | 2021 | Associations between tolerance toward foreign residents and the big five personality traits in Japan: The moderating effect of socioecological variable | Yoshino, S., Oshio, A. |
|  | 2021 | Achieving multiple socio-ecological institutional fits: The case of spiny lobster co-management in Wagu, Japan | Ishihara, H., et al. |
|  | 2021 | The Influence of Socio-Ecological Networks on Willingness to Communicate in English for Japanese People | Ito, T. |
| Kazakhstan | 2019 | Water, dust, and agro-pastoralism: Modeling socio-ecological co-evolution of landscapes, farming, and human society in southeast Kazakhstan during the mid to late Holocene | Ullah, I.I.T., et al. |
| Luxembourg | 2021 | Can citizen science complement official data sources that serve as evidence-base for policies and practice to improve water quality? | König, A., et al. |
| Netherlands | 2012 | Dairy farming and newt habitat: How shocks in milk prices influence the optimal design of water retention policies | Cormont, A., et al. |
|  | 2016 | Modelling socio-ecological systems with MAIA: A biogas infrastructure simulation | Verhoog, R., et al. |
|  | 2017 | New Interest in Wild Forest Products in Europe as an Expression of Biocultural Dynamics | Wiersum, K.F. |
|  | 2018 | Facilitating energy transition through energy commons: An application of socio-ecological systems framework for integrated community energy systems | Acosta, C., et al.. |
|  | 2020 | Impact of student interventions on urban greening processes | Stobbelaar, D.J. |
|  | 2021 | Resilient Drinking Water Resources | Kloosterman, R.A., et al. |
| New Zealand | 2009 | Sustainable Water allocation for families, fish and farming: A wicked problem or a wicked solution? | Painter, B.D.M. |
|  | 2010 | Farmer models of socio-ecologic systems: Application of causal mapping across multiple locations | Fairweather, J. |
|  | 2013 | An integrated biophysical and socio-economic framework for analysis of climate change adaptation strategies: The case of a New Zealand dairy farming system | Kalaugher, E., et al. |
|  | 2016 | Key biocultural values to guide restoration action and planning in New Zealand | Phil O’B. Lyver, et al. |
|  | 2017 | Using environmental report cards to monitor implementation of iwi plans and strategies, including restoration plans | Tipa, G.T., et al. |
|  | 2018 | Can sustainability auditing be indigenized? | Reid, J., Rout, M. |
|  | 2018 | Human Perceptions of Megafaunal Extinction Events Revealed by Linguistic Analysis of Indigenous Oral Traditions | Wehi, P.M., et al. |
|  | 2018 | Collaborative freshWater planning: changing roles for science and scientists | Berkett, N., et al. |
|  | 2018 | Translating Ecological Integrity terms into operational language to inform societies | de Juan, S., et al. |
|  | 2019 | Characterising resilience in the wine industry: Insights and evidence from Marlborough, New Zealand | Cradock-Henry, N.A., Fountain, J. |
|  | 2019 | The importance of connected ocean monitoring knowledge systems and communities | Kaiser, B.A., et al. |
|  | 2019 | Biodiver_Cities: An exploration of how architecture and urban design can regenerate ecosystem services | Koat, J., Zari, M.P. |
|  | 2020 | Psychological resilience, organizational resilience and life satisfaction in tourism firms: insights from the Canterbury earthquakes | Prayag, G., et al. |
|  | 2020 | A landscape and landscape biography approach to assessing the consequences of an environmental policy implementation | Spicer, E.A., et al. |
|  | 2020 | Coastal tectonics and habitat squeeze: response of a tidal lagoon to co-seismic sea-level change | Orchard, S., et al. |
|  | 2021 | Good predators: The roles of weka (gallirallus australis) in new zealand’s past and present ecosystems | Carpenter, J.K., et al. |
| Norway | 2012 | Community-Based Management: Under What Conditions Do Sámi Pastoralists Manage Pastures Sustainably? | Hausner, V.H., et al. |
|  | 2013 | Marine angling tourism in Norway and Iceland: Finding balance in management policy for sustainability | Solstrand, M.-V. |
|  | 2014 | Something fishy: Assessing stakeholder resilience to increasing jellyfish (Periphylla periphylla) in Trondheimsfjord, Norway | Gjelsvik Tiller, R., et al. |
|  | 2015 | Mapping value plurality towards ecosystem services in the case of Norwegian wildlife management: A Q analysis | Bredin, Y.K., et al. |
|  | 2015 | Institutional challenges for effective governance of consumptive wildlife tourism: case studies of marine angling tourism in Iceland and Norway | Solstrand, M.-V. |
|  | 2016 | Pasture access and adaptive capacity in reindeer herding districts in Nordland, Northern Norway | Risvoll, C., Hovelsrud, G.K. |
|  | 2016 | Stakeholder perceptions of links between environmental changes to their socio-ecological system and their adaptive capacity in the region of Troms, Norway | Tiller, R., et al. |
|  | 2019 | Ceased grazing management changes the ecosystem services of semi-natural grasslands | Johansen, L., et al. |
| Russia | 2013 | Methodology of socially-oriented observations and the possibilities of their implementation in the Arctic resilience assessment | Vlasova, T., Volkov, S. |
|  | 2018 | Measurement of Public Interest in Ecological Matters Through Online Activity and Environmental Monitoring | Verzilin, D., et al. |
|  | 2018 | Spatial Heterogeneity of Russia in the Light of the Concept of a Green Economy: The Social Context | Glazyrina, I.P., Zabelina, I.A. |
|  | 2019 | Approaches to the sustainability assessment of Water resources and their applicability to the arctic rivers | Shestakova, E., Fedorova, I. |
|  | 2019 | Energy of reindeer breeding: Driving actions in the controlling impacts in ecological and ethno-social contexts | Klokov, K.B. |
|  | 2019 | Statistical methods in the analysis of pollution impacts on human health | Rossinskaya, M.V., et al. |
|  | 2019 | The problem of Water and sanitation on the example of India and Russia | Osadchuk, M.A., et al. |
|  | 2020 | Collecting and Processing Distributed Data for Decision Support in Social Ecology | Verzilin, D., et al. |
|  | 2020 | Network platform for tourism sector: Transformation and interpretation of multifaceted data | Kuklina, M., et al. |
|  | 2020 | Mortality trends in the population of the Irkutsk region in the process of social and environmental transformations (1989-2017) | Leshchenko, Y.A., Lisovtsov, A.A. |
|  | 2021 | The river Alazeya: Shifting Socio-Ecological Systems Connected to a Northeastern Siberian river | Mustonen, T., Shadrin, V. |
| South Africa | 2007 | Linking ecosystem services and Water resources: Landscape-scale hydrology of the Little Karoo | Le Maitre, D.C., et al. |
|  | 2007 | Ecosystems services in South Africa: a research theme that can engage environmental, economic and social scientists in the development of sustainability science? | Le Maitre, D.C., et al. |
|  | 2007 | Seeking common ground: How natural and social scientists might jointly create an overlapping worldview for sustainable livelihoods: A South African perspective | King, N., et al. |
|  | 2011 | The next decade of environmental science in South Africa: A horizon scan | Shackleton, C.M., et al. |
|  | 2011 | New perspectives in small-scale fisheries management: Challenges and prospects for implementation in South Africa | Sowman, M. |
|  | 2011 | Strategic Adaptive Management in freshWater protected areas and their rivers | Kingsford, R.T., et al. |
|  | 2012 | Social Learning Through Environmental Risk Analysis of Biodiversity and GM Maize in South Africa | Dana, G.V., Nelson, K.C. |
|  | 2012 | Water management institutions for more resilient societies | Muller, M. |
|  | 2012 | Moving from traditional government to new adaptive governance: The changing face of food security responses in South Africa | Pereira, L.M., Ruysenaar, S. |
|  | 2013 | The future of the food system: Cases involving the private sector in South Africa | Pereira, L.M. |
|  | 2013 | Analysing risk and vulnerability of South African settlements: Attempts, explorations and reflections | van Huyssteen, E., et al. |
|  | 2013 | The race for space: Tracking land-cover transformation in a socio-ecological landscape, South Africa | Coetzer, K.L., et al. |
|  | 2013 | Urban ecology in a developing world: Why advanced socioecological theory needs Africa | McHale, M.R., et al. |
|  | 2014 | Elevated elephant density does not improve ecotourism opportunities: Convergence in social and ecological objectives | Maciejewski, K., Kerley, G.I.H. |
|  | 2015 | The role of regional climate projections in managing complex socio-ecological systems | Daron, J.D., et al. |
|  | 2016 | Another look at economic approaches to environmental management and policy with reference to developments in South Africa editorial | de Wit, M. |
|  | 2016 | Shifting from Individual to Collective Action: Living Land's experience in the Baviaanskloof, South Africa | Talbot, M., van den Broeck, D. |
|  | 2016 | Expert-derived monitoring thresholds for impacts of megaherbivores on vegetation cover in a protected area | Smit, I.P.J., et al. |
|  | 2016 | Cooperation and collapse in a communal livestock production SES model - A case from South Africa | Rasch, S., et al. |
|  | 2016 | Reorganizing resource use in a communal livestock production socio-ecological system in South Africa | Rasch, S., et al. |
|  | 2016 | Thresholds of change in a multi-use conservation landscape of South Africa: Historical land-cover, future transformation and consequences for environmental decision-making | Coetzer-Hanack, K.L., et al. |
|  | 2017 | Multi-scale resilience of a communal rangeland system in South Africa | Rasch, S., et al. |
|  | 2017 | Putting Spatial Resilience into Practice | Barnes, A., Nel, V. |
|  | 2017 | Aiming for the biodiversity target with the social welfare arrow: medicinal and other useful plants from a Critically Endangered grassland ecosystem in Limpopo Province, South Africa | Dzerefos, C.M., et al. |
|  | 2017 | Modelling informal Sand Forest harvesting using a Disturbance Index from Landsat, in Maputaland (South Africa) | Nel, R., et al. |
|  | 2019 | A changing fishery system: perspectives from crew in the Southern Cape’s handline fishery | Gammage, L.C., et al. |
|  | 2019 | Ecosystem services and ecological degradation of communal wetlands in a South African biodiversity hotspot | Pantshwa, A.O., Buschke, F.T. |
|  | 2020 | Rapid games designing; constructing a dynamic metaphor to explore complex systems and abstract concepts | Lankford, B.A., Craven, J. |
|  | 2020 | A new protocol for monitoring operational outcomes of environmental management in commercial forestry plantations | Joubert-van der Merwe, L., et al. |
|  | 2020 | The current status of indigenous ovine genetic resources in Southern Africa and future sustainable utilisation to improve livelihoods | Molotsi, A.H., et al. |
|  | 2020 | Editorial overview: recommendations for the promotion of a resilient linefishery in the Anthropocene | Potts, W.M., et al. |
|  | 2021 | Nexus planning as a pathway towards sustainable environmental and human health post Covid-19 | Nhamo, L., Ndlela, B. |
| South Korea | 2020 | Exploring community symbiotic tourism programs for the utilization and conservation of ecology in lava stony forest (Gotjawal) of Jeju Island, Korea | Hong, C.-Y., et al. |
|  | 2021 | Development of landscape conservation value map of Jeju island, Korea for integrative landscape management and planning using conservation value of landscape typology | Jun, B., et al. |
| Spain | 1992 | Environmental exploitation and social structure in prehistoric southeast Spain | Ruiz, M., et al. |
|  | 2008 | Recreation suitability analysis: Application in protected and non-protected areas | De Aranzabal, I., et al. |
|  | 2010 | Social multi-criteria evaluation as a decision support tool for integrated coastal zone management | Garmendia, E., et al. |
|  | 2012 | Methodology to assess sustainable management of water resources in coastal lagoons with agricultural uses: An application to the Albufera lagoon of Valencia (Eastern Spain) | Usaquén Perilla, O.L., et al. |
|  | 2012 | Managing structural uncertainty for sustainability: A case study from Monegros, Spain | Bernal, E., Zografos, C. |
|  | 2013 | Sustainability and social-ecological resilience in the Ebro delta | Romagosa, F., et al. |
|  | 2013 | Temporal Changes in Socio-Ecological Systems and Their Impact on Ecosystem Services at Different Governance Scales: A Case Study of Heathlands | Morán-Ordóñez, A., et al. |
|  | 2013 | Participatory process to prioritize actions for a sustainable management in a biosphere reserve | Onaindia, M., et al. |
|  | 2013 | The Spanish livestock model: A coevolutionary analysis | Ríos-Núñez, S.M., et al. |
|  | 2014 | Community, common-pool resources and socio-ecological systems: Water management and community building in southern Spain | Ruiz-Ballesteros, E., Gálvez-García, C. |
|  | 2014 | Positive and negative feedbacks and free-scale pattern distribution in rural-population dynamics | Alados, C.L., et al. |
|  | 2015 | "Us" and "them". Fishermen from Gandía and the loss of institutional legitimacy | Herrera-Racionero, P., et al. |
|  | 2015 | Dynamic integration of sustainability indicators in insular socio-ecological systems | Banos-González, I., et al. |
|  | 2015 | Bayesian Estimation Dating of Lithic Surface Collections | Fernández-López de Pablo, J., Barton, C.M. |
|  | 2015 | Analysis of the socioecological structure and dynamics of the territory using a hybrid Bayesian network classifier | Ropero, R.F., et al. |
|  | 2015 | Conservation Traps and Long-Term Species Persistence in Human-Dominated Systems | Cardador, L., et al.. |
|  | 2016 | Main aspects for integrated coastal zone management in Spain: Concepts, terminology, context, and delimitation criteria | Barragán, J.M., De Andrís, M. |
|  | 2016 | Widening the analysis of Energy Return on Investment (EROI) in agro-ecosystems: Socio-ecological transitions to industrialized farm systems (the Vallès County, Catalonia, c.1860 and 1999) | Galán, E., et al.. |
|  | 2016 | Is “socio-ecological culture” really being taken into account to manage conflicts in the coastal zone? Inputs from Spanish Mediterranean beaches | Ariza, E., et al. |
|  | 2017 | Structure of Stockmen Collaboration Networks under Two Contrasting Touristic Regimes in the Spanish Central Pyrenees | Saiz, H., et al. |
|  | 2017 | Integrating knowledge exchange and the assessment of dryland management alternatives – A learning-centered participatory approach | Bautista, S., et al. |
|  | 2017 | Defining adaptation measures collaboratively: A participatory approach in the Doñana socio-ecological system, Spain | De Stefano, L., et al. |
|  | 2018 | Identifying socio-ecological networks in rural-urban gradients: Diagnosis of a changing cultural landscape | Arnaiz-Schmitz, C., et al. |
|  | 2018 | Exploring multi-dimensional recreational quality of beach socio-ecological systems in the Canary Islands (Spain) | Peña-Alonso, C., et al. |
|  | 2018 | Probabilistic modeling of the relationship between socioeconomy and ecosystem services in cultural landscapes | Maldonado, A.D., et al. |
|  | 2018 | The Integrated Territorial Investment (ITI) of the Mar Menor as a model for the future in the comprehensive management of enclosed coastal seas | Garcia-Ayllon, S. |
|  | 2019 | Is circular economy the key to transitioning towards sustainable development? Challenges from the perspective of care ethics | Pla-Julián, I., Guevara, S. |
|  | 2019 | The opaque lagoon. Water management and governance in L’albufera de València Wetland (Spain) | Jégou, A., Sanchis-Ibor, C. |
|  | 2019 | New strategies to improve co-management in enclosed coastal seas and wetlands subjected to complex environments: Socio-economic analysis applied to an international recovery success case study after an environmental crisis | García-Ayllón, S. |
|  | 2019 | Regional fire scenarios in Spain: Linking landscape dynamics and fire regime for wildfire risk management | Montiel Molina, C., et al. |
|  | 2019 | The building of a management system for marine recreational fisheries in Galicia (NW Spain) | Pita, P., Villasante, S. |
|  | 2019 | A double-loop process for beach quality index construction: Approaching the complexity of the Catalan coast | Bombana, B., Ariza, E. |
|  | 2019 | In the blind-spot of governance – Stakeholder perceptions on seagrasses to guide the management of an important ecosystem services provider | Ruiz-Frau, A., et al. |
|  | 2019 | Social attributes can drive or deter the sustainability of bottom-up management systems | Rivera, A., et al. |
|  | 2020 | Place attachment, feeling of belonging and collective identity in socio-ecological systems: Study case of pegalajar (Andalusia-Spain) | Escalera-Reyes, J. |
|  | 2020 | The Use of Recreational Fishers’ Ecological Knowledge to Assess the Conservation Status of Marine Ecosystems | Pita, P., et al. |
|  | 2020 | Understanding conservation conflicts associated with rodent outbreaks in farmland areas | Lauret, V., et al. |
|  | 2020 | Where do ecosystem services come from? Assessing and mapping stakeholder perceptions on water ecosystem services in the muga river basin (catalonia, spain) | Garau, E., et al. |
|  | 2021 | Can agriculture and conservation be compatible in a coastal wetland? Balancing stakeholders’ narratives and interactions in the management of El Hondo Natural Park, Spain | Ricart, S., Rico-Amorós, A.M. |
|  | 2021 | An integral approach to address socio-ecological systems sustainability and their uncertainties | Martínez-Fernández, J., et al. |
|  | 2021 | The natural assurance value of nature-based solutions: A layered institutional analysis of socio ecological systems for long term climate resilient transformation | López Gunn, E., et al. |
|  | 2021 | Water for food, water for birds: How to manage conflicting rural-natural interfaces? Deepening on the socio-ecological system of El Hondo Natural Park (Alicante, Spain) | Ricart, S., Rico-Amorós, A.M. |
|  | 2021 | Follow the flow: Analysis of relationships between water ecosystem service supply units and beneficiaries | Garau, E., et al. |
|  | 2021 | Depopulation impacts on ecosystem services in Mediterranean rural areas | Bruno, D., et al. |
| Sweden | 2006 | Reaching for new perspectives on socio-ecological systems: Exploring the possibilities for adaptive co-management in the Swedish mountain region | Willebrand, T., et al. |
|  | 2011 | Colonial tutelage and industrial colonialism: Reindeer husbandry and early 20th-century hydroelectric development in Sweden | Ossbo, Å., Lantto, P. |
|  | 2015 | Closing the collaborative gap: Aligning social and ecological connectivity for better management of interconnected wetlands | Kininmonth, S., et al. |
|  | 2015 | A handshake between markets and hierarchies: Geese as an example of successful collaborative management of ecosystem services | Tuvendal, M., Elmberg, J. |
|  | 2017 | The importance of socio-ecological system dynamics in understanding adaptation to global change in the forestry sector | Blanco, V., et al. |
|  | 2018 | Capturing complexity: Forests, decision-making and climate change mitigation action | Klapwijk, M.J., et al.. |
|  | 2021 | Interrelated Factors for Return to Work of Sick-Listed Employees in Sweden | Selander, J., et al. |
| Switzerland | 2013 | Balancing control and complexity in field studies of neonicotinoids and honey bee health | Suryanarayanan, S. |
|  | 2014 | Organising for socio-ecological resilience: The roles of the mountain farmer cooperative genossenschaft gran alpin in graubünden, switzerland | Bardsley, D.K., Bardsley, A.M. |
|  | 2021 | Discovering the wild side of urban plants through public engagement | Vega, K.A., et al. |
| Turkey | 2017 | ‘Stream Daylighting’ as an approach for the renaturalization of riverine systems in urban areas: Istanbul-Ayamama Stream case | Delibas, M., Tezer, A. |
|  | 2018 | Not the End of the World? Post-Classical Decline and Recovery in Rural Anatolia | Roberts, N., et al. |
| United Kingdom | 2000 | Ecological Networks: Connecting environmental, economic and social systems? | James, P., et al. |
|  | 2009 | Land use and the state of the natural environment | Potschin, M. |
|  | 2010 | Navigability and the improvement of the river Thames, 1605-1815 | Oliver, S. |
|  | 2013 | Liquid materialities in the landscape of the Thames: Mills and weirs from the eighth century to the nineteenth century | Oliver, S. |
|  | 2013 | How resilient are europe's inshore fishing communities to change? Differences between the north and the south | Hadjimichael, M., et al. |
|  | 2014 | Coastal zone ecosystem services: From science to values and decision making; a case study | Luisetti, T., et al. |
|  | 2016 | Of resilient places: planning for urban resilience | Mehmood, A. |
|  | 2019 | Modelling land use dynamics in socio-ecological systems: A case study in the UK uplands | Termansen, M., et al. |
|  | 2019 | To what extent has sustainable intensification in England been achieved? | Armstrong McKay, D.I., et al. |
|  | 2019 | Identifying barriers, conflict and opportunity in managing aquatic ecosystems | Robinson, L.A., et al. |
|  | 2019 | How are nature based solutions contributing to priority societal challenges surrounding human well-being in the United Kingdom: A systematic map protocol | Dick, J., et al. |
|  | 2020 | Stakeholder perceptions of public good provision from agriculture and implications for governance mechanism design | Roberts, M., et al. |
| United States | 1999 | Coarse woody debris in riparian zones: Opportunity for interdisciplinary interaction | Bragg, D.C., Kershner, J.L. |
|  | 2006 | Resilience lost: Intersecting land use and landscape dynamics in the prehistoric southwestern United States | Peeples, M.A., et al. |
|  | 2006 | A graduate education framework for tropical conservation and development | Kainer, K.A., et al. |
|  | 2007 | Linking ecosystem health indicators and collaborative management: A systematic framework to evaluate ecological and social outcomes | Muñoz-Erickson, T.A., et al. |
|  | 2009 | Regimes of information: Land use, management, and policy | Ekbia, H.R., Evans, T.P. |
|  | 2009 | Collectively engaging complex socio-ecological systems: re-envisioning science, governance, and the California Delta | Norgaard, R.B., et al. |
|  | 2010 | Climate change adaptation strategies for federal forests of the Pacific Northwest, USA: Ecological, policy, and socio-economic perspectives | Spies, T.A., et al. |
|  | 2011 | Promoting wellness in Alaskan villages: Integrating traditional knowledge and science of wild berries | Flint, C.G., et al. |
|  | 2012 | Ecosystem processes and human influences regulate streamflow response to climate change at long-term ecological research sites | Jones, J.A., et al. |
|  | 2012 | Don't fence me in: Boundaries, policy, and deliberation in Maine's lobster commons | Brewer, J.F. |
|  | 2012 | Uncovering the spatial dynamics of wild rice Lakes, harvesters and management across Great Lakes landscapes for shared regional conservation | Drewes, A.D., Silbernagel, J. |
|  | 2012 | Heterogeneity in Residential Yard Care: Evidence from Boston, Miami, and Phoenix | Harris, E.M., et al. |
|  | 2012 | Efficiency Through Proximity: Changes in Phosphorus Cycling at the Urban-Agricultural Interface of a Rapidly Urbanizing Desert Region | Metson, G., et al. |
|  | 2013 | What are we protecting out here? A political ecology of forest, fire, and fuels management in utah's wildland-urban interface | Roberts, J. |
|  | 2013 | Balancing control and complexity in field studies of neonicotinoids and honey bee health | Suryanarayanan, S. |
|  | 2013 | Simulating effects of land use policies on extent of the wildland urban interface and wildfire risk in Flathead County, Montana | Paveglio, T.B., et al. |
|  | 2013 | Understanding and managing marine protected areas through integrating ecosystem based management within maritime cultural landscapes: Moving from theory to practice | Barr, B.W. |
|  | 2014 | Classification of Watersheds into integrated social and biophysical indicators with clustering analysis | Mayer, A., et al. |
|  | 2014 | Adaptive capacity in light of Hurricane Sandy: The need for policy engagement | Wagner, M., et al. |
|  | 2014 | The role of reserves and anthropogenic habitats for functional connectivity and resilience of ephemeral wetlands | Uden, D.R., et al. |
|  | 2014 | Using uncertainty and sensitivity analyses in socioecological agent-based models to improve their analytical performance and policy relevance | Ligmann-Zielinska, A., et al. |
|  | 2015 | Predictors, spatial distribution, and occurrence of woody invasive plants in subtropical urban ecosystems | Staudhammer, C.L., et al. |
|  | 2015 | "Communities in the middle": Interactions between drivers of change and place-based characteristics in rural forest-based communities | Morzillo, A.T., et al. |
|  | 2015 | Applying a socio-ecological framework to thematic analysis using a statewide assessment of disproportionate minority contact in the United States | Henderson, D.X., Baffour, T. |
|  | 2015 | Risk management is not enough: a conceptual model for resilience and adaptation-based vulnerability assessments | Sikula, N.R., et al. |
|  | 2015 | The Unintended Ecological and Social Impacts of Food Safety Regulations in California's Central Coast Region | Karp, D.S., et al. |
|  | 2016 | Sustainable Remediation of Legacy Mine Drainage: A Case Study of the Flight 93 National Memorial | Emili, L.A., et al. |
|  | 2016 | Using landscape typologies to model socioecological systems: Application to agriculture of the United States Gulf Coast | Surendran Nair, S., et al. |
|  | 2016 | Early warning signals of social transformation: A case study from the US southwest | Spielmann, K.A., et al. |
|  | 2017 | Discerning and addressing environmental failures in policy scenarios using Planning Support System (PSS) technologies | Deal, B., Pan, H. |
|  | 2017 | Why watch bees? Motivations of citizen science volunteers in the Great Pollinator Project | Domroese, M.C., Johnson, E.A. |
|  | 2017 | The Socioecology of Sexual and Reproductive Health Care Use Among Young Urban Minority Males | Marcell, A.V., et al. |
|  | 2017 | Development and testing a diagnostic capacity tool for improving socio-ecological system governance | McKay, P.A., et al. |
|  | 2017 | Historical and projected trends in landscape drivers affecting carbon dynamics in Alaska | Pastick, N.J., et al. |
|  | 2017 | An adaptable agent-based model for guiding multi-species Pacific salmon fisheries management within a SES framework | Cenek, M., Franklin, M. |
|  | 2017 | Critical dynamics in population vaccinating behavior | Pananos, A.D., et al. |
|  | 2018 | Angler travel distances: Implications for spatial approaches to marine recreational fisheries governance | Camp, E.V., et al. |
|  | 2018 | Measuring urban tree loss dynamics across residential landscapes | Ossola, A., Hopton, M.E. |
|  | 2018 | Land Manager Perceptions of Opportunities and Constraints of Using Livestock to Manage Invasive Plants | Shapero, M.W.K., et al. |
|  | 2018 | Climate differentiates forest structure across a residential macrosystem | Ossola, A., Hopton, M.E. |
|  | 2018 | Interdependencies and Risk to People and Critical Food, Energy, and Water Systems: 2013 Flood, Boulder, Colorado, USA | Romero-Lankao, P., Norton, R. |
|  | 2018 | Perceptions, Experiences, and Priorities Supporting Agroecosystem Management Decisions Differ among Agricultural Producers, Consultants, and Researchers | McKenzie, S., et al. |
|  | 2019 | Transgender and Other Gender-Diverse Youth’s Progression Through the HIV Continuum of Care Socioecological System barriers | Harper, G.W., et al. |
|  | 2019 | A review of coastal management approaches to support the integration of ecological and human community planning for climate change | Powell, E.J., et al. |
|  | 2019 | Characterizing and comparing marine fisheries ecosystems in the United States: determinants of success in moving toward ecosystem-based fisheries management | Link, J.S., Marshak, A.R. |
|  | 2019 | Assessing local attitudes and perceptions of non-native species to inform management of novel ecosystems | Lewis, C.L., et al. |
|  | 2019 | Quantifying irrigation adaptation strategies in response to stakeholder-driven ground Water management in the US High Plains Aquifer | Deines, J.M., et al. |
|  | 2019 | A dynamic and spatially explicit modeling approach to identify the ecosystem service implications of complex urban systems interactions | Pan, H., et al. |
|  | 2019 | Northern forest winters have lost cold, snowy conditions that are important for ecosystems and human communities | Contosta, A.R., et al. |
|  | 2019 | Yards increase forest connectivity in urban landscapes | Ossola, A., et al. |
|  | 2019 | Winter Weather Whiplash: Impacts of Meteorological Events Misaligned With Natural and Human Systems in seasonally Snow-Covered Regions | Casson, N.J., et al. |
|  | 2019 | Human-carnivore relations: Conflicts, tolerance and coexistence in the American West | Expósito-Granados, M., et al. |
|  | 2020 | Global social and environmental change drives the management and delivery of ecosystem services from urban gardens: A case study from Central Coast, California | Lin, B.B., Egerer, M.H. |
|  | 2020 | Adaptive rangeland management benefits grassland birds utilizing opposing vegetation structure in the shortgrass steppe | Davis, K.P., et al. |
|  | 2020 | Pathways of organisational transformation for sustainability: a university case-study synthesis presenting competencies for systemic change & rubrics of transformation | Baker-Shelley, A., et al. |
|  | 2020 | ‘Push through everything’: using phenomenological inquiry to investigate how Black males’ socioecology and identities promote perseverance in the U. S. public education system | Henderson, D.X., et al. |
|  | 2020 | Assessing improvements in socio-ecological system governance using mixed methods and the quality governance framework and its diagnostic capacity tool | McKay, P.A., et al. |
|  | 2020 | Linking yard plant diversity to homeowners’ landscaping priorities across the U.S | Padullés Cubino, J., et al. |
|  | 2020 | Giant Sequoia—Forest, Monument, or Park?: Political-Legal Mandates and Socio-Ecological Complexity Shaping Landscape-Level Management | Jenkins, J., Brown, M. |
|  | 2020 | Conserving biodiversity takes a plan: How planners implement ecological information for biodiversity conservation | Gagné, S.A., et al. |
|  | 2020 | Holistic perspectives—Understanding rancher experiences with holistic resource management to bridge the gap between rancher and researcher perspectives | Barton, E., et al. |
|  | 2020 | Observation-derived ice growth curves show patterns and trends in maximum ice thickness and safe travel duration of Alaskan Lakes and rivers | Arp, C.D, et al. |
|  | 2020 | Marsh Migration, Climate Change, and Coastal Resilience: Human Dimensions Considerations for a Fair Path Forward | Van Dolah, E.R., et al. |
|  | 2021 | Exploring the use of ecosystem services conceptual models to account for the benefits of public lands: An example from national forest planning in the United States | Olander, L., et al. |
|  | 2021 | Implications of Zoonoses From Hunting and Use of Wildlife in North American Arctic and Boreal Biomes: Pandemic Potential, Monitoring, and Mitigation Implications of Zoonoses From Hunting and Use of Wildlife in North American Arctic and Boreal Biomes: Pandemic Potential, Monitoring, and Mitigation | Keatts, L.O., et al. |
|  | 2021 | Climate change, resilience, and the Native American fisher-hunter-gatherers of the late Holocene on the Georgia coast, USA | Ritchison, B.T., et al.. |
|  | 2021 | COVID-19 as Eco-Pandemic Injustice: Opportunities for Collective and Antiracist Approaches to Environmental Health | Powers, M., et al. |
|  | 2021 | Blending Ecosystem Service and Resilience Perspectives in Planning of Natural Infrastructure: Lessons from the San Francisco Bay Area | Hamel, P., et al. |
|  | 2021 | The Chesapeake Bay program modeling system: Overview and recommendations for future development | Hood, R.R., et al. |
| Uruguay | 2014 | Predation of South American sea lions (*Otaria flavescens*) on artisanal fisheries in the Rio de la Plata estuary | De María, M., et al. |
|  | 2020 | Commoning the management of the socio-ecological system of the Pampa biome: Analysis of a collective of traditional ranchers from Uruguay | Severo, C.M., Matte, A. |

**Annex 3.** Socio-ecological knowledge exchange in terms of the number of papers published between the analysed countries in both hemispheres, where the origin was the first author's affiliation country, and the destination was the country where the research was conducted.

| **Origin** | **Destination** | ***n* papers** |
| --- | --- | --- |
| Argentina | Chile | 1 |
| Australia | South Africa | 1 |
| Australia | United States | 3 |
| Brazil | Uruguay | 1 |
| Canada | Australia | 1 |
| Canada | Denmark | 1 |
| Canada | Japan | 1 |
| Canada | Turkey | 1 |
| Canada | United States | 2 |
| Chile | Spain | 1 |
| China | Hong Kong | 1 |
| Denmark | Chile | 1 |
| Denmark | New Zealand | 1 |
| Finland | Russia | 1 |
| France | Belgium | 1 |
| France | Germany | 1 |
| France | Spain | 2 |
| Germany | Argentina | 1 |
| Germany | Georgia | 1 |
| Germany | South Africa | 3 |
| Greece | France | 1 |
| Italy | Argentina | 1 |
| Italy | Canada | 1 |
| Japan | Canada | 1 |
| Netherlands | Australia | 1 |
| Netherlands | Chile | 1 |
| Netherlands | Germany | 1 |
| Netherlands | United States | 1 |
| Norway | Denmark | 1 |
| Spain | Argentina | 1 |
| Spain | Chile | 1 |
| Spain | New Zealand | 1 |
| Spain | United States | 1 |
| Switzerland | France | 1 |
| Switzerland | Germany | 1 |
| Switzerland | Netherlands | 1 |
| Taiwan | Japan | 1 |
| United Kingdom | Argentina | 1 |
| United Kingdom | Chile | 1 |
| United Kingdom | Iceland | 1 |
| United Kingdom | South Africa | 2 |
| United Kingdom | Sweden | 1 |
| United States | Botswana | 6 |
| United States | Chile | 3 |
| United States | Kazakhstan | 1 |
| United States | South Africa | 3 |
| United States | Spain | 1 |
| No knowledge exchange |  | 404 |
| Total |  | 467 |
